# Manipulation of dorsal raphe serotonergic neurons modulates active coping to inescapable stress and anxiety-related behaviors in mice and rats

**DOI:** 10.1038/s41386-018-0254-y

**Published:** 2018-10-30

**Authors:** Naoya Nishitani, Kazuki Nagayasu, Nozomi Asaoka, Mayumi Yamashiro, Chihiro Andoh, Yuma Nagai, Haruko Kinoshita, Hiroyuki Kawai, Norihiro Shibui, Beihui Liu, James Hewinson, Hisashi Shirakawa, Takayuki Nakagawa, Hitoshi Hashimoto, Sergey Kasparov, Shuji Kaneko

**Affiliations:** 1 Department of Molecular Pharmacology, Graduate School of Pharmaceutical Sciences, Kyoto University, 46-29 Yoshida-Shimoadachi-cho, Sakyo-ku, Kyoto 606-8501 Japan; 2 Drug Innovation Center, Graduate School of Pharmaceutical Sciences, Osaka University, 1-6 Yamadaoka, Suita, Osaka 565-0871 Japan; 3 Center for the Promotion of Interdisciplinary Education and Research, Kyoto University, Yoshidahommachi, Sakyo-ku, Kyoto 606-8501 Japan; 4 School of Physiology and Pharmacology, University of Bristol, Bristol, UK; 5 Department of Clinical Pharmacology and Therapeutics, Kyoto University Hospital, 54 Shogoin Kawahara-cho, Sakyo-ku, Kyoto 606-8507 Japan; 6 Laboratory of Molecular Neuropharmacology, Graduate School of Pharmaceutical Sciences, Osaka University, 1-6 Yamadaoka, Suita, Osaka 565-0871 Japan; 7 Molecular Research Center for Children’s Mental Development, United Graduate School of Child Development, Osaka University, Kanazawa University, Hamamatsu University School of Medicine, Chiba University and University of Fukui, 1-6 Yamadaoka, Suita, Osaka 565-0871 Japan

**Keywords:** Gene delivery, Optogenetics, Stress and resilience

## Abstract

Major depression and anxiety disorders are a social and economic burden worldwide. Serotonergic signaling has been implicated in the pathophysiology of these disorders and thus has been a crucial target for pharmacotherapy. However, the precise mechanisms underlying these disorders are still unclear. Here, we used species-optimized lentiviral vectors that were capable of efficient and specific transduction of serotonergic neurons in mice and rats for elucidation of serotonergic roles in anxiety-like behaviors and active coping behavior in both species. Immunohistochemical analyses revealed that lentiviral vectors with an upstream sequence of tryptophan hydroxylase 2 gene efficiently transduced serotonergic neurons with a specificity of approximately 95% in both mice and rats. Electrophysiological recordings showed that these lentiviral vectors induced sufficient expression of optogenetic tools for precise control of serotonergic neurons. Using these vectors, we demonstrate that acute activation of serotonergic neurons in the dorsal raphe nucleus increases active coping with inescapable stress in rats and mice in a time-locked manner, and that acute inhibition of these neurons increases anxiety-like behaviors specifically in rats. These findings further our understanding of the pathophysiological role of dorsal raphe serotonergic neurons in different species and the role of these neurons as therapeutic targets in major depression and anxiety disorders.

## Introduction

Major depression and anxiety disorders are large-scale medical and societal problems worldwide [1]. Although a variety of neurotransmitters and stress hormones have been implicated [2–7], serotonergic neurons are generally believed to play the central role in the pathogenesis and thus the pharmacotherapy of these disorders. Specifically, clinical studies have repeatedly demonstrated that selective serotonin reuptake inhibitors (SSRIs) are effective for treatment of major depression, at least in part of the patients [8–10]. Consistent with this notion, most antidepressants increase the extracellular serotonin concentration in the brain [11]. Previous reports in mice have demonstrated that antidepressant-like effects of SSRIs as well as ketamine depend on the serotonin system [12, 13]. Besides, serotonin deficiency induced by genetic deletion or by an inactivating mutation of tryptophan hydroxylase 2 (TPH2), a rate-limiting enzyme for serotonin synthesis in the brain, increases depression-like behavior in mice [14, 15]. In addition, chemogenetic approaches have revealed that stimulation of central serotonergic neurons elicits antidepressant-like effect in mice [16]. However, it is well known that serotonergic neurons are heterogeneous in terms of their connectivity and function [17, 18]. In this context, it is still unclear which subgroups of serotonergic neurons are responsible for antidepressant action of serotonin in the brain.

Mice with genetic deletion of TPH2 exhibited a complex phenotype, whereby elevated plus maze suggested lower level of anxiety, while forced swim test revealed increased immobility and the aggressive behavior was greatly increased in these knockout mice [14]. In a different model with an inactivating mutation of TPH2, increased anxiety-like behaviors have been documented [19]. On the other hand, chemogenetic approach has revealed that activation of central serotonergic neurons increases anxiety-like behaviors in mice [16]. In human, SSRIs and buspirone, a partial agonist of 5HT_1A_ receptor, have been widely used for treatment of anxiety disorders, indicating that modulation of serotonergic neural transmission decreases anxiety [20, 21]. However, in human patients SSRIs induce “activation syndrome”, manifesting as irritability, agitation, anxiety, and other undesirable similar emotional states, during the initial phase of its treatment [22]. Therefore, it is still unclear whether and how central serotonergic neurons affect the level of anxiety.

Optogenetics has been widely used to control neuronal activity with high spatial and temporal resolution [23]. In previous studies, the technique has been applied using transgenic mice, in which the activity of genetically targeted serotonergic neurons can be controlled [24–28]. These studies demonstrated that stimulation of serotonergic neurons increases anxiety [26, 28], promotes waiting for rewards [24, 27], and reinforces instrumental learning [25], while the consequences of optogenetic inhibition of these neurons remain to be elucidated. Furthermore, it is well known that there are major differences in animal behavior between mice strains and even more so between the species [29, 30]. Therefore, it is important to use comparable methods to investigate consequences of activation and inhibition of serotonergic system in other species such as rat. This requires a tool for potent and highly specific expression of optogenetic constructs enabling cell-type-specific optogenetic control in the desired locations. In this context, TPH-Cre transgenic rats have been used for specific labeling of serotonergic neurons in rats [31]. The behavioral consequences of their optogenetic/chemogenetic manipulation, however, have not yet been examined. We previously described viral vectors with two-step transcriptional amplification (TSTA) that make use of GAL4-p65 chimeric protein and GAL4 binding sequence and are capable of potent gene expression under cell-type-specific promoters such as glial fibrillary acidic protein and TPH2, in rats [32, 33]. Here, we developed new species-optimized self-inactivating lentiviral vectors (LVV) capable of optogenetic control of serotonergic neurons in mice and rats. We then tested whether specific activation or suppression of the serotonergic neurons in the dorsal raphe nucleus (DRN) modulates mood-related behaviors.

## Materials and methods

### Animals and stereotaxic surgery

All animal care and experimental procedures were in accordance with the ethical guidelines of the Kyoto University Animal Research Committee. The adult male C57BL/6J mice (6−9 weeks old, Nihon SLC, Shizuoka, Japan) and Wistar/ST male rats (2−3 weeks old (electrophysiology), 6−9 weeks old (behavioral experiment), Nihon SLC) were housed in groups (no more than 6 mice/3 rats in an individual cage) with free access to food and water, and kept under constant ambient temperature (24 ± 1 °C) and humidity (55 ± 10%), with a 12-h light−dark cycles. Animals were randomly assigned to each experimental group.

The details of stereotaxic surgeries are described in Supplementary Materials and Methods.

### Production and purification of self-inactivating lentiviral vector

Production and purification of self-inactivating LVV [34] were performed as described previously [35]. The details of LVV production were described in Supplementary Materials and Methods.

### In vivo optogenetic manipulation

The fiber-optic cannulae were made of multimode LC/PC ceramic ferrules (1.25 mm outer diameter, 270 µm hole size, Thorlabs, Newton, NJ, USA) and plastic optic fiber (CK10, 250 µm diameter, NA 0.5, Mitsubishi Rayon, Tokyo, Japan). The fiber-optic cannula implanted to mice were connected to the fiber-optic patch cord (Doric Lenses, Quebec, Canada) coupled with the rotary joint (Doric Lenses). Light emitted from the diode-pumped solid-state (DPSS) laser (Beijing Viasho Technology, Beijing, China) was converged to the fiber optic by the FC/PC collimator (Thorlabs), which was connected to the rotary joint. The DPSS laser was driven by the electric stimulator (Nihon Kohden, Tokyo, Japan).

For srTPH2:ChETA rats, srTPH2:Venus controls, smTPH2:ChETA mice, and smTPH2:Venus controls, blue light illumination (473 nm, 20 mW at the tip of the fiber, 5 ms duration, 20 Hz frequency) was delivered from 15 min before the test to the end of the test session (Figs. 2, 4) or between minutes 3 to 6 of the behavioral tests (Figs. 3, 5). In Figs. 2 and 4, optogenetic stimulation was initiated before the behavioral test to mimic the effect of pretreatment with an antidepressant. For srTPH2:eArchT rats, srTPH2:Venus controls, smTPH2:eArchT mice, and smTPH2:Venus controls, green light illumination (532 nm, 10 mW at the tip of the fiber, continuous) was delivered throughout the test session (Figs. 2, 4) or between minutes 3 to 6 of the behavioral tests (Figs. 3, 5). In Fig. 4, half of the srTPH2:Venus-injected rats were illuminated with blue light and another half were illuminated with green light. Because there was no significant difference between these two groups in any of the behavioral tests performed, we considered these two groups as one control group (shown as srTPH2:Venus in Fig. 4).

### Behavioral tests

All behavioral tests were performed and analyzed by experimenters who were blind to injected LVV. The animals with misplaced fiber-optic cannula were excluded from the analysis. All behavioral tests were performed during the light phase of the day cycle.

#### Experimental design

The time course of behavioral experiments was described in Supplementary Figure S1.

In Fig. 2, two independent cohorts of mice were used, where cohort 1 underwent the tail suspension test and cohort 2 underwent the open field test (day 1) and the elevated plus maze test (day 2). In Fig. 4, one cohort of rats underwent the open field test (day 1), the elevated plus maze test (day 2), and the forced swim test (day 3). In Figs. 3, 5, Supplementary Figures S13, S14, S15, two independent cohort of the animals were used as follows: In mice, cohort 1 underwent the light−dark transition test, the open field test, the elevated plus maze test, and the tail suspension test. Cohort 2 underwent social interaction test and real-time place preference test. In rats, cohort 1 underwent the light−dark box test, the open field test, the elevated plus maze test and the forced swim test. Cohort 2 underwent two-chamber real-time place preference test. All behavioral tests were performed in the order as indicated above, and at least 24 h apart.

#### Tail suspension test

All experiments were performed 1−2 weeks after viral injection and 1−2 days after an implantation of the fiber-optic cannula. The tail suspension test was performed as previously described [36] with brief modifications for light delivery. Briefly, the fiber-optic patch cord was plugged into the fiber-optic cannula preimplanted into the mice. After acclimation, the mice were hung on a hook (35 cm from the floor of the test box) with the tail taped to a force-transducer (PowerLab 2/26, AD Instruments, Dunedin, New Zealand) fixed to the ceiling of the test box (40 × 40 × 40 cm). The immobility time was recorded for 6 min (Fig. 2) or for 9 min (Fig. 3). In Supplementary Figure S8, administration of citalopram (LKT Laboratories, St. Paul, MN, USA) was performed 30 min before testing. In all other experiments, no drug injections were performed. The behavior of mice was video-recorded throughout the test, and the mice that held their hindlimbs or climbed their tails with their forelimbs during the tail suspension test were excluded from the analysis.

#### Open field test

The open field arena consisting of a white acrylic cube (rats: 75 × 75 × 40 cm, mice: 50 × 50 × 50 cm) was divided into a center zone (rats: 35 × 35 cm, mice: 25 × 25 cm) and an outer zone in the periphery. Each animal was connected to the fiber-optic patch cord, placed individually into the center of the arena and permitted free exploration. The behavior of the animal was recorded with a camera over a 5-min (rats) or a 10-min (mice) session (Figs. 2, 4) or a 9-min session (Figs 3, 5); the recorded data were analyzed automatically using video tracking system (ANY-maze version 4.99, Stoelting, Wood Dale, IL, USA). Total distance traveled and time spent in center zone during a session were measured.

#### Elevated plus maze test

The elevated plus maze test was performed 1 day after the open field test. The apparatus consisted of two open arms and two closed arms (rats: 60 × 10 cm, mice: 30 × 5 cm) extended from a central platform (rats: 10 × 10 cm, mice: 5 × 5 cm). Each animal was plugged into the fiber-optic patch cord and placed individually into the central platform. The behavior of the animal was recorded with a camera over a 5-min (rats) or a 10-min (mice) session (Figs. 2, 4) or 9-min session (Figs. 3, 5); the recorded data were analyzed automatically using video tracking system (ANY-maze version 4.99). Time spent in the open and closed arms and total distance traveled during a session were measured. Animals that fell during the test were excluded from the analysis.

#### Forced swim test

The forced swim test was performed after the elevated plus maze test. On the first day of the test, rats were individually placed in a clear cylinder with water (20 cm in diameter, 25 cm depth, 25 ± 1 °C) for 15 min as pre-swim session. On the next day, each rat was plugged into the fiber-optic patch cord and was allowed to swim for 10 min. The sessions were recorded from the top of the cylinder and the recorded data were analyzed by experimenter who are blind to injected LVV. Mobility was defined as vertical movement of the forepaws and horizontal movement.

The details of light−dark transition test, social interaction test, and real-time place preference test are described in Supplementary Materials and Methods.

### Histology

The details of histological analysis are described in Supplementary Materials and Methods.

### Electrophysiology

Electrophysiological analysis was performed as described in a previous report [36]. The details of electrophysiological analysis are described in Supplementary Materials and Methods.

### Vector construction

The sequences of oligodeoxynucleotide primers (Hokkaido System Science, Sapporo, Japan) were summarized in Supplementary Table 1. PCR was performed with Phusion or Q5 DNA polymerase (New England Biolabs, Ipswich, MA, USA). All ligation reaction was performed with T4 DNA ligase (BioAcademia, Osaka, Japan). The details of vector construction are described in Supplementary Materials and Methods. The plasmids used in this study will be available upon request.

### Statistical analysis

Statistical analysis was performed by GraphPad Prism 5 (GraphPad Software Inc., La Jolla, CA, USA). Two-sided unpaired Student’s *t* test was used for comparisons of two individual groups unless otherwise stated. One-way or two-way ANOVA followed by Bonferroni post hoc test was used for group comparisons unless otherwise stated. Difference was considered significant at *P* < 0.05. For parametric test, data distribution was assumed to be normal but this was not formally tested. The variance within each group was analyzed by F-test or Bartlett test. The variance is similar between groups in all figures except Figs. 2e (Time in Center), 4b (Time in Center) and 4c. Thus, Fig. 2e (Time in Center) was analyzed by *t* test with Welch’s correction, and Fig. 4b (Time in Center) and 4c were analyzed by Kruskal−Wallis test with Dunn’s Multiple Comparison test.

## Results

### Improved-TSTA lentiviral vectors are capable of optogenetic control of serotonergic neurons

**Fig. 1:**
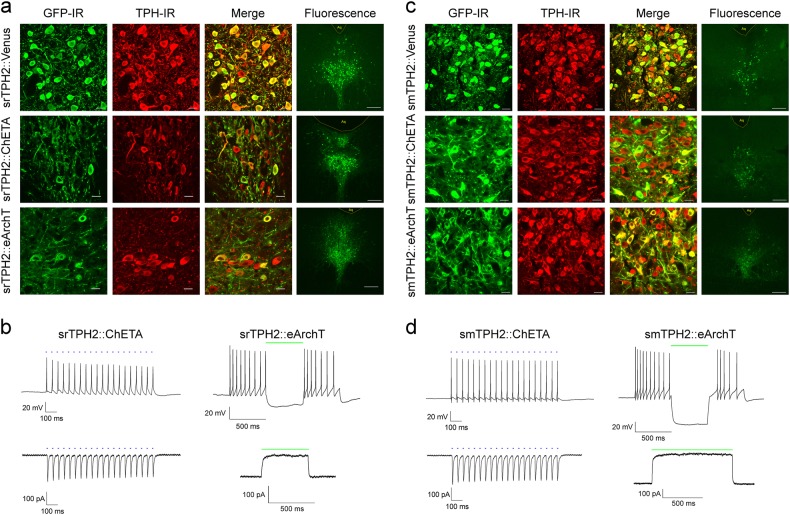
srTPH2 and smTPH2 LVVs efficiently transduced serotonergic neurons in the DRN. **a**, **b** Rats were stereotaxically injected with srTPH2:Venus, srTPH2:ChETA or srTPH2:eArchT in the DRN. **a** One week after injection, coronal sections containing the DRN were prepared and stained by anti-GFP and anti-TPH antibodies. Stained sections were imaged using confocal microscopy. Some sections were left unstained in order to assess the expression levels of the transgenes (Venus or eYFP). Representative images from at least three rats are shown. Scale bars = 20 μm (GFP, TPH, Merge) or 200 μm (Fluorescence). **b** One week after injection of srTPH2:ChETA or srTPH2:eArchT, 200-μm-thick coronal slices were prepared. The cells with eYFP fluorescence were analyzed by whole cell patch clamp. Blue light (20 pulses, 20 Hz, 5 ms duration) or green light (1 pulse, 0.5 s duration) was applied through fiber optics to illuminate patched cells. Representative traces of current-clamp recording (upper panel) and voltage-clamp recording (lower panel) are shown. Similar results were obtained in two (ChETA) and three (eArchT) rats. **c**, **d** Mice were injected with smTPH2:Venus, smTPH2:ChETA, or smTPH2:eArchT in the DRN. **c** One week after injection, coronal sections were prepared and processed as described in (**a**). Representative images from at least three mice are shown. Scale bars = 20 μm (GFP, TPH, Merge) or 200 μm (Fluorescence). **d** One week after injection of smTPH2:ChETA or smTPH2:eArchT, 200-μm-thick coronal slices were prepared. The cells with eYFP fluorescence were analyzed by whole cell patch clamp. Blue light (20 pulses, 20 Hz, 5 ms duration) or green light (1 pulse, 0.5 s or 1 s duration) was applied through fiber optics to illuminate patched cells. Representative traces of current-clamp recording (upper panel) and voltage-clamp recording (lower panel) were shown. Similar results were obtained in eight (ChETA) and three (eArchT) mice

The previously described LVV with TSTA and rat TPH2 promoter [33] (rTPH2-Venus) induced only modest expression of EGFP variant Venus which was detectable only after immunohistochemical enhancement in the rat DRN (Supplementary Figure S2, S3). The key step in TSTA is the binding of the chimeric transcription factor GAL4-p65 upstream of the TPH2 promoter. To increase gene expression we modified this LVV so that GAL4-p65 chimeric transcription factor was in-frame to 11th ATG of upstream IRES for more efficient GAL4-p65 expression [37]. We also placed an mRNA stabilizing sequence of the woodchuck hepatitis virus posttranscriptional regulatory element (WPRE) into the 3′UTR of the cassette thus generating (rTPH2-Venus-WPRE) (Supplementary Figure S2, S3). We then replaced Venus with either ChETA-eYFP or eArchT-eYFP fusions [38, 39]. To evaluate the suitability of the modified LVVs for optogenetic manipulation and to assess their cell-type specificity, we injected rTPH2-Venus-WPRE (srTPH2:Venus), rTPH2-ChETA-eYFP-WPRE (srTPH2:ChETA), or rTPH2-eArchT-eYFP-WPRE (srTPH2:eArchT) into the DRN in rats (Fig. 1). One week after LVV injection, 96.9 ± 0.9% (obtained from three rats) of Venus immunoreactive cells were TPH immunoreactive in the DRN (Supplementary Figure S4), and fluorescence of Venus and eYFP were detectable without immunohistochemical enhancement (Fig. 1a, Supplementary Figure S3). In acute DRN slice prepared from srTPH2:ChETA-injected rats, 473 nm light illumination (5 ms pulses at 20 Hz) induced action potentials and evoked inward currents in eYFP-positive cells (Fig. 1b). In contrast, in acute DRN slice from srTPH2:eArchT-injected rats, continuous green light (532 nm) illumination evoked outward current and suppressed action potential generation induced by current injection (100 pA) in eYFP-positive cells (Fig. 1b). These results indicate that the modified LVVs are capable of inducing sufficient gene expression for optogenetic control of serotonergic neurons.

Even though many promoters operate cross-species, this was not the case with the rat TPH2 promoter which was not sufficiently effective in our hands. Therefore to apply these LVVs to mice, we replaced rat TPH2 promoter with its mouse ortholog, creating smTPH2:Venus, smTPH2:ChETA, and smTPH2:eArchT. One week after injection of the LVVs into the DRN in mice, fluorescence of Venus- or eYFP-positive cells was detectable without immunohistochemical enhancement whereas 94.0 ± 0.9% (obtained from three mice) of Venus immunoreactive cells were immunoreactive for TPH (Fig. 1c, Supplementary Figure S5). Blue light illumination (20 Hz) of eYFP-positive cells faithfully induced inward currents and evoked action potentials in the slice from smTPH2:ChETA-injected mice (Fig. 1d), and continuous green light illumination induced outward current and completely suppressed action potential generation evoked by current injection in slices from smTPH2:eArchT-injected mice (Fig. 1d). These results indicate that the modified LVVs with species-specific promoters drive expression of optogenetic actuators sufficiently to manipulate serotonergic neurons in rats and mice with high cell-type specificity. To test whether blue light application increases serotonegic activity not only ex vivo but also in vivo, we examined c-fos expression, a marker for neuronal activity, after optogenetic manipulation. Blue light illumination (20 Hz) for 3 min via optic fiber placed above the DRN increased c-Fos expression in eYFP- and TPH2-double-positive cells in srTPH2:ChETA, but not in the srTPH2:Venus in rats. Moreover, blue light illumination (20 Hz) for 3 min increased c-Fos expression in eYFP- and TPH2-double-positive cells in smTPH2:ChETA, but not in the smTPH2:Venus in mice (Supplementary Figure S6). This result indicates that the modified LVVs are capable of activation of serotonergic neurons in vivo.

### Activation of DRN serotonergic neurons promotes active coping to stress in mice

**Fig. 2:**
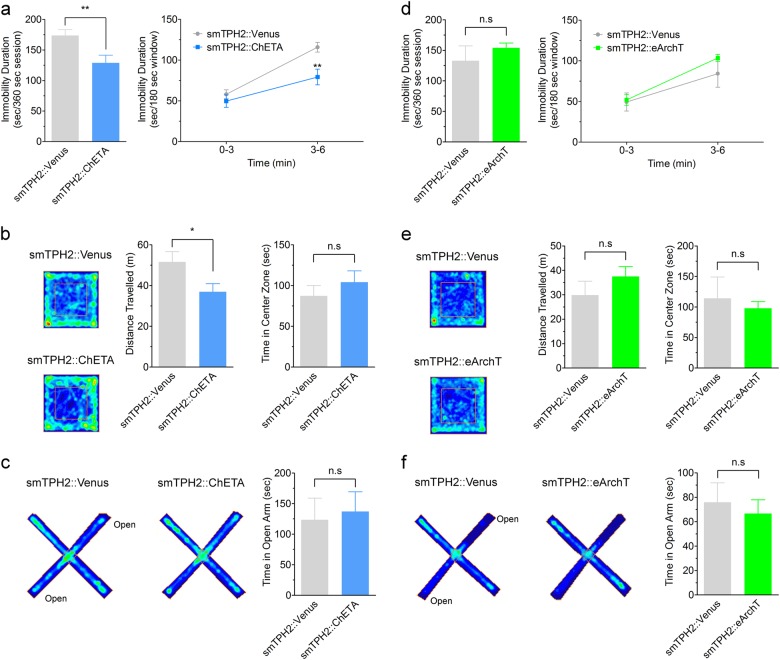
Specific stimulation of the DRN serotonergic neurons induces antidepressant-like effect in mice. **a** One week after injection of smTPH2:ChETA or smTPH2:Venus in the DRN, immobility duration in the tail suspension test was examined in the presence of blue light illumination to the DRN. Blue light was applied at 20 Hz with 5 ms duration from 15 min before the test to the end of the test session. Data represent mean ± SEM of immobility duration throughout the test session (left) or in first and second half of the test session (right). *n* = 12 (Venus), 10 (ChETA) mice. **P < 0.01 vs. Venus (left: Venus, 173.8 ± 9.5 s, ChETA, 128.9 ± 12.6 s; unpaired *t* test; *P* = 0.009; right: Venus 0−3, 58.0 ± 5.4 s, Venus 3−6, 115.8 ± 6.0 s, ChETA 0−3, 49.6 ± 7.7 s, ChETA 3−6, 79.2 ± 9.6 s; two-way ANOVA; Interaction, *F*(1, 20) = 4.79, *P* = 0.041, Time, *F*(1, 20) = 45.98, *P* < 0.0001, Opto, *F*(1, 20) = 8.38, *P* = 0.009; Bonferroni posttests; 0−3, *P* > 0.05, 3−6, *P* < 0.01). **b** Representative heat map analysis of one animal in each group (left). One week after injection of smTPH2:ChETA or smTPH2:Venus in the DRN, distance traveled (center) and time in center zone (right) in the open field test were examined in the presence of blue light illumination to the DRN. Blue light was applied at 20 Hz with 5 ms duration from 15 min before the test to the end of the test session. Data represent mean ± SEM. *n* = 8 (Venus), 7 (ChETA) mice. *P < 0.05 vs. Venus. n.s.: not significant (distance traveled: Venus, 51.6 ± 5.1 m, ChETA, 36.9 ± 4.0 m; unpaired *t* test; *P* = 0.046, time in center zone: Venus, 87.4 ± 12.7 s, ChETA, 104.2 ± 13.8 s; unpaired *t* test; *P* = 0.384). **c** One day after the open field test, time in open arms in the elevated plus maze test was examined in the presence of blue light illumination to the DRN. Blue light was applied at 20 Hz with 5 ms duration from 15 min before the test to the end of the test session. Representative heat map analysis of one animal in each group (left). Data represent mean ± SEM. *n* = 8 (Venus), 7 (ChETA) mice (right). n.s.: not significant (Venus, 123.5 ± 35.5 s, ChETA, 137.2 ± 32.3 s; unpaired *t* test; *P* = 0.782). **d** One week after injection of smTPH2:eArchT or smTPH2:Venus in the DRN, immobility duration in the tail suspension test was examined in the presence of green light illumination to the DRN. Continuous green light was applied throughout the test session. Data represent mean ± SEM of immobility duration throughout the test session (left) or in the former and latter half of the test session (right). *n* = 7 (Venus), 9 (eArchT) mice. n.s.: not significant (left: Venus, 132.9 ± 24.6 s, eArchT, 153.9 ± 8.0 s; unpaired *t* test; *P* = 0.382; right: Venus 0−3, 49.4 ± 11.1 s, Venus 3−6, 84.2 ± 16.8 s, eArchT 0−3, 51.9 ± 6.6 s, eArchT 3−6, 103.4 ± 4.4 s; two-way ANOVA; Interaction, *F*(1, 14) = 1.34, *P* = 0.266, Time, *F*(1, 14) = 36.17, *P* < 0.0001, Opto, *F*(1, 14) = 0.82, *P* = 0.380; Bonferroni posttests; 0−3, *t*(14) = 0.182, *P* > 0.05, 3−6, *t*(14) = 1.374, *P* > 0.05). **e** Representative heat map analysis of one animal in each group (left). One week after injection of smTPH2:eArchT or smTPH2:Venus in the DRN, distance traveled (center) and time in center zone (right) in the open field test (600 s) were examined in the presence of green light illumination to the DRN. Continuous green light was applied throughout the test session. Data represent mean ± SEM. *n* = 5 (Venus), 7 (eArchT) mice. n.s.: not significant (distance traveled: Venus, 29.9 ± 5.7 m, eArchT, 37.5 ± 4.0 m; unpaired *t* test; *P* = 0.281, time in center zone: Venus, 114.1 ± 35.0 s, eArchT, 98.0 ± 10.1 s; unpaired *t* test with Welch’s correction; *P* = 0.682). **f** One day after the open field test, time in open arms in the elevated plus maze test (600 s) was examined in the presence of green light illumination to the DRN. Continuous green light was applied throughout the test session. Representative heat map analysis of one animal in each group (left). Data represent mean ± SEM. *n* = 6 (Venus), 8 (eArchT) mice (right). n.s.: not significant (Venus, 75.9 ± 16.1 s, eArchT, 53.3 ± 9.8 s; unpaired *t* test; *P* = 0.256)

To assess whether activation of serotonergic neurons in the DRN is sufficient to induce antidepressant-like effect, we performed the tail suspension test in mice 1 week after injection of smTPH2:ChETA in the DRN (Fig. 2a, Supplementary Figure S7, S8). This test assesses animal’s immobility as a proxy of feeling of helplessness. In the presence of blue light stimulation, mice injected with smTPH2:ChETA showed significantly less immobility duration in the second half (3−6 min) of the session compared to smTPH2:Venus-injected mice (Fig. 2a). Interestingly, acute administration of an SSRI, citalopram (2, 4, 8 mg/kg, i.p.), significantly decreased the immobility duration throughout the test session in a dose-dependent manner (Supplementary Figure S9). To rule out the possibility that this decreased immobility duration is due to a general increase in locomotor activity, we performed the open field test. However, mice injected with smTPH2:ChETA had significantly less locomotor activity compared to smTPH2:Venus-expressing mice in the presence of blue light stimulation (Fig. 2b). These results demonstrate that acute selective stimulation of the DRN serotonergic neurons is able to induce an antidepressant-like effect in mice.

Various lines of evidences also indicate serotonin in the control of anxiety [4, 14, 16, 19–21]. However, in our experiments the time spent in the center zone of the open field was similar in mice injected with smTPH2:ChETA and smTPH2:Venus in the presence of blue light stimulation (Fig. 2b). In another anxiety test, the elevated plus maze test, the exploration time in the open arm of smTPH2:ChETA-injected mice was also not significantly different from that of smTPH2:Venus-injected animals (Fig. 2c). These results indicate that acute selective stimulation of the DRN serotonergic neurons is not sufficient for inducing anxiolytic (or anxiogenic) effect even though it clearly antagonizes passive coping behavior.

Next, we investigated the effect of selective suppression of the DRN serotonergic neurons by using smTPH2:eArchT. We found that the immobility in the tail suspension test in smTPH2:eArchT-injected mice was similar to that of smTPH2:Venus-injected mice in the presence of green light illumination (Fig. 2d, Supplementary Figure S10). This result suggests that selective acute suppression of the DRN serotonergic neurons is not sufficient to induce prodepressive-like effect. Furthermore, both locomotor activity and the exploration time in center in the open field test of the mice injected with smTPH2:eArchT were similar to those of smTPH2:Venus-injected mice in the presence of green light (Fig. 2e). Moreover, in the elevated plus maze test, there was no significant difference in the exploration time in the open arm of smTPH2:eArchT-injected mice and that of smTPH2:Venus-injected mice in the presence of green light illumination (Fig. 2f). Collectively, these data suggest that selective suppression of the DRN serotonergic neurons is not sufficient to affect locomotor activity and the level of anxiety.

**Fig. 3:**
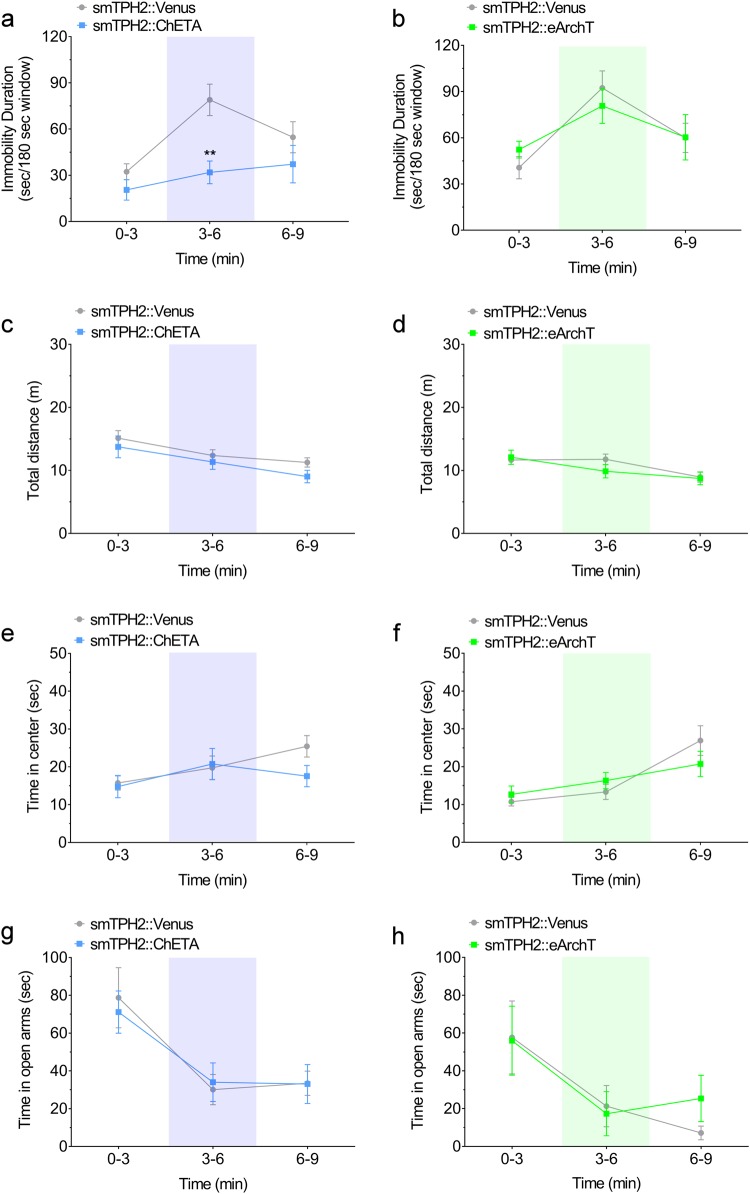
Effect of transient activation of mouse DRN serotonergic neurons on stress-coping and anxiety-related behaviors. **a**, **b** One week after injection of smTPH2:ChETA, smTPH2:eArchT, or smTPH2:Venus in the DRN, the effect of transient light stimulation of DRN on immobility in the tail suspension test was examined. Light was applied from 3 to 6 min of the test. Data represent mean ± SEM of immobility duration in each time window of the test session. *n* = 8 (Venus in **a**), 9 (ChETA), 11 (Venus in **b**), 8 (eArchT) mice. **P < 0.01 vs. Venus (**a** Venus 0−3, 32.3 ± 5.2 s, 3−6, 78.9 ± 10.2 s, 6−9, 54.7 ± 10.1 s, ChETA 0−3, 20.5 ± 6.6 s, 3−6, 31.9 ± 7.4 s, 6−9, 37.2 ± 12.1 s; two-way ANOVA; Interaction, *F*(2, 30) = 3.61, *P* = 0.040, Time, *F*(2, 30) = 8.81, *P* = 0.001, Opto, *F*(1, 15) = 6.82, *P* = 0.020; Bonferroni posttests; 0−3, *P* > 0.05, 3−6, *P* < 0.01, 6−9, *P* > 0.05, **b** Venus 0−3, 40.7 ± 7.3 s, 3−6, 92.4 ± 11.0 s, 6−9, 60.0 ± 9.5 s, eArchT 0−3, 52.3 ± 5.4 s, 3−6, 80.7 ± 11.4 s, 6−9, 60.4 ± 14.7 s; two-way ANOVA; Interaction, *F*(2, 34) = 1.12, *P* = 0.338, Time, *F*(2, 34) = 13.56, *P* < 0.0001, Opto, *F*(1, 17) = 0.00014, *P* = 0.991; Bonferroni posttests; 0−3, *P* > 0.05, 3−6, *P* > 0.05, 6−9, *P* > 0.05). **c**−**f** One week after injection of smTPH2:ChETA, smTPH2:eArchT, or smTPH2:Venus in the DRN, open field test was performed in the presence of light illumination to the DRN. Light was applied from 3 to 6 min of the test session. Data represent mean ± SEM of traveled distance or spent time in the center zone in each time window of the test session. *n* = 9 (Venus in **c**, **e**), 10 (ChETA), 11 (Venus in **d**, **f**), 10 (eArchT) mice. No significant difference was observed (**c** Venus 0−3, 15.1 ± 1.2 m, 3−6, 12.4 ± 0.9 m, 6−9, 11.3 ± 0.7 m, ChETA 0−3, 13.7 ± 1.7 m, 3−6, 11.3 ± 1.2 m, 6−9, 9.0 ± 1.0 m; two-way ANOVA; Interaction, *F*(2, 34) = 0.45, *P* = 0.639, Time, *F*(2, 34) = 21.63, *P* < 0.001, Opto, *F*(1, 17) = 1.06, *P* = 0.319; Bonferroni posttests; 0−3, *P* > 0.05, 3−6, *P* > 0.05, 6−9, *P* > 0.05, **d** Venus 0−3, 11.7 ± 0.7 m, 3−6, 11.8 ± 0.8 m, 6−9, 9.0 ± 0.8 m, eArchT 0−3, 12.1 ± 1.1 m, 3−6, 9.9 ± 1.1 m, 6−9, 8.7 ± 1.0 m; two-way ANOVA; Interaction, *F*(2, 38) = 1.62, *P* = 0.212, Time, *F*(2, 38) = 10.51, *P* = 0.0002, Opto, *F*(1, 19) = 0.294, *P* = 0.594; Bonferroni posttests; 0−3, *P* > 0.05, 3−6, *P* > 0.05, 6−9, *P* > 0.05, **e** Venus 0−3, 15.7 ± 1.9 s, 3−6, 19.7 ± 3.1 s, 6−9, 25.4 ± 2.8 s, ChETA 0−3, 14.8 ± 2.9 s, 3−6, 20.8 ± 4.1 s, 6−9, 17.6 ± 2.8 s; two-way ANOVA; Interaction, *F*(2, 34) = 2.76, *P* = 0.077, Time, *F*(2, 34) = 5.52, *P* = 0.008, Opto, *F*(1, 17) = 0.499, *P* = 0.490; Bonferroni posttests; 0−3, *P* > 0.05, 3−6, *P* > 0.05, 6−9, *P* > 0.05, **f** Venus 0−3, 10.8 ± 1.1 s, 3−6, 13.3 ± 2.0 s, 6−9, 26.9 ± 3.9 s, eArchT 0−3, 12.7 ± 2.2 s, 3−6, 16.3 ± 2.1 s, 6−9, 20.7 ± 3.3 s; two-way ANOVA; Interaction, *F*(2, 38) = 2.91, *P* = 0.067, Time, *F*(2, 38) = 18.17, *P* < 0.0001, Opto, *F*(1, 19) = 0.022, *P* = 0.885; Bonferroni posttests; 0−3, *P* > 0.05, 3−6, *P* > 0.05, 6−9, *P* > 0.05). **g**, **h** One week after injection of smTPH2:ChETA, smTPH2:eArchT, or smTPH2:Venus in the DRN, elevated plus maze test was performed in the presence of light illumination to the DRN. Light was applied from 3 to 6 min of the test session. Data represent mean ± SEM of spent time in the open arms in each time window of the test session. *n* = 9 (Venus in **g**), 10 (ChETA), 11 (Venus in **h**), 10 (eArchT) mice. No significant difference was observed (**g** Venus 0−3, 78.7 ± 15.9 s, 3−6, 30.1 ± 8.0 s, 6−9, 33.5 ± 6.4 s, ChETA 0−3, 71.1 ± 11.2 s, 3−6, 34.0 ± 10.2 s, 6−9, 33.1 ± 10.3 s; two-way ANOVA; Interaction, *F*(2, 34) = 0.197, *P* = 0.822, Time, *F*(2, 34) = 13.9, *P* < 0.0001, Opto, *F*(1, 17) = 0.016, *P* = 0.901; Bonferroni posttests; 0−3, *P* > 0.05, 3−6, *P* > 0.05, 6−9, *P* > 0.05, **h** Venus 0−3, 57.7 ± 19.3 s, 3−6, 21.3 ± 10.9 s, 6−9, 7.2 ± 3.6 s, eArchT 0−3, 56.0 ± 18.3 s, 3−6, 17.3 ± 11.7 s, 6−9, 25.4 ± 12.3 s; two-way ANOVA; Interaction, *F*(2, 38) = 0.886, *P* = 0.421, Time, *F*(2, 38) = 12.1, *P* < 0.0001, Opto, *F*(1, 19) = 0.068, *P* = 0.798; Bonferroni posttests; 0−3, *P* > 0.05, 3−6, *P* > 0.05, 6−9, *P* > 0.05)

To investigate whether promotion of active coping in mice by DRN serotonergic neurons is time-locked, we examined the effect of transient optogenetic stimulation/inhibition in the middle of behavioral analysis. We found that optogenetic stimulation of the mouse DRN serotonergic neurons from 3 to 6 min of the tail suspension session significantly decreased the immobility duration of this time window, but by 9 min, the effect of stimulation was lost (Fig. 3a). On the other hand, optogenetic inhibition of these neurons in the middle of the tail suspension session did not affect the immobility throughout the experiment (Fig. 3b). We also performed the open field test and elevated plus maze test with transient light illumination (Fig. 3c−h, Supplementary Figures S11, S12). There were no significant difference in total distance traveled (Fig. 3c, d) and time spent in the center zone (Fig. 3e, f). Similarly in the elevated maze transient stimulation or inhibition in the middle of the test were inefficient (Fig. 3g, h). Furthermore, we investigated the effect of optogenetic activation/inhibition of DRN serotonergic neurons in light−dark transition test, another behavioral paradigm examining anxiety. However we found no significant effect with optogenetic activation and inhibition on the spent time in the dark area, an index of anxiety-like state (Supplementary Figure S13a, b). Moreover, we examined the effect of optogenetic activation/inhibition of DRN serotonergic neurons in real-time place preference test and social interaction test, for investigating valence and sociality, respectively. There were no significant difference in the spent time in the area associated with laser stimulation in the real-time place preference test (Supplementary Figure S14a, b), and the spent time in the interaction zone in the social interaction test (Supplementary Figure S15-17). These results indicate that stimulation of DRN serotonergic neurons rapidly promotes active coping to stress.

### Rat DRN serotonergic neurons promote coping with stress and decrease anxiety

**Fig. 4:**
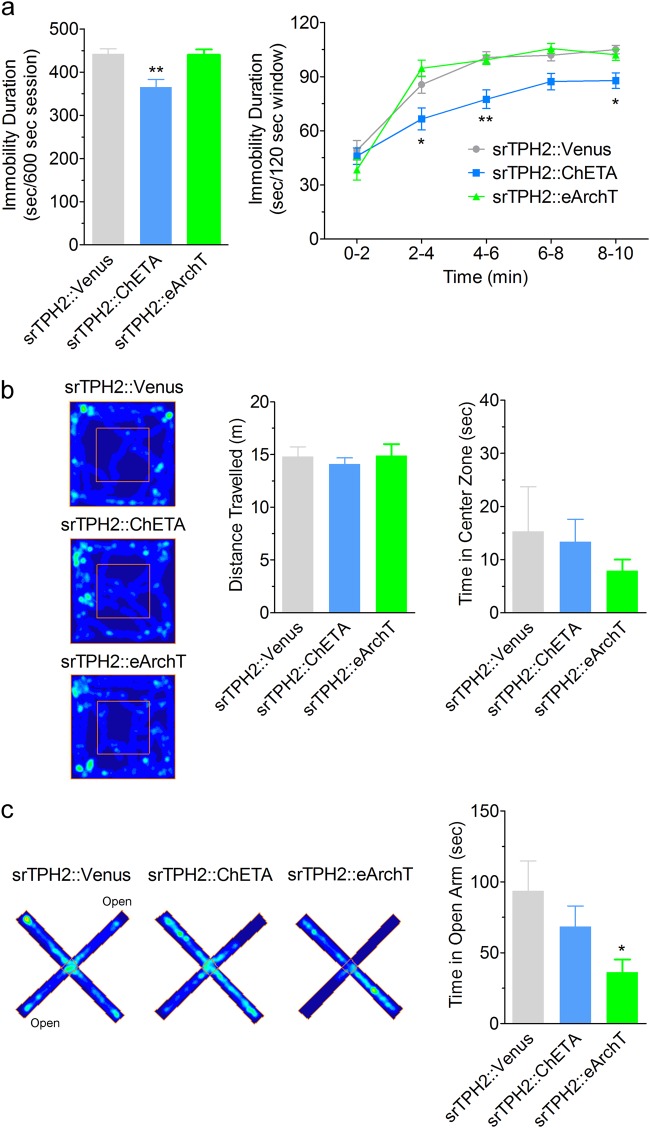
Effects of specific manipulation of the DRN serotonergic neurons on mood-related behaviors in rats. **a** After injection of srTPH2:Venus, srTPH2:ChETA, or srTPH2:eArchT in the DRN, the open field test and the elevated plus maze test, immobility duration in the forced swim test was examined in the presence of light illumination to the DRN. Data represent mean ± SEM of immobility duration throughout the test session (left) or in each 2-min window of the test session (right). *n* = 11 (Venus), 13 (ChETA), 10 (eArchT) rats. *P < 0.05, **P < 0.01 vs. Venus (Left: Venus, 442.5 ± 11.9 s, ChETA, 365.2 ± 18.1 s, eArchT, 440.2 ± 12.5 s; one-way ANOVA; *F*(2, 31) = 8.96, *P* = 0.0009; Bonferroni’s Multiple Comparison Test; Venus vs. ChETA, *P* < 0.01, Venus vs. eArchT, *P* > 0.05, ChETA vs. eArchT, *P* < 0.01; Right: Venus, 49.2 ± 5.5 s (0−2), 85.6 ± 4.8 s (2−4), 100.7 ± 3.2 s (4−6), 101.9 ± 3.1 s (6−8), 105.1 ± 2.3 s (8−10), ChETA, 46.0 ± 4.6 s (0−2), 66.6 ± 6.1 s (2−4), 77.5 ± 5.1 s (4−6), 87.3 ± 4.6 s (6−8), 87.8 ± 4.3 s (8−10), eArchT, 38.4 ± 5.8 s (0−2), 94.7 ± 4.5 s (2−4), 99.4 ± 2.6 s (4−6), 105.6 ± 2.7 s (6−8), 102.1 ± 3.0 s (8−10); two-way ANOVA; Interaction, *F*(8, 124) = 3.70, *P* = 0.0007, Time, *F*(4, 124) = 112.50, *P* < 0.0001, Opto, *F*(2, 124) = 8.96, *P* = 0.0009; Bonferroni posttests; Venus vs. ChETA 0−2, *P* > 0.05, 2−4, *P* < 0.05, 4−6, *P* < 0.01, 6−8, *P* > 0.05, 8−10, *P* < 0.05, Venus vs. eArchT 0−2, *P* > 0.05, 2−4, *P* > 0.05, 4−6, *P* > 0.05, 6−8, *P* > 0.05, 8−10, *P* > 0.05). **b** Representative heat map analysis of one animal in each group (left). One week after injection of srTPH2:Venus, srTPH2:ChETA, or srTPH2:eArchT in the DRN, distance traveled (center) and time in center zone (right) in the open field test (300 s) were examined in the presence of light illumination to the DRN. Data represent mean ± SEM. *n* = 11 (Venus), 13 (ChETA), 12 (eArchT) rats. There was no significant difference between groups (Left: Venus, 14.8 ± 0.9 m, ChETA, 14.1 ± 0.6 m, eArchT, 14.9 ± 1.1 m; one-way ANOVA; *F*(2, 33) = 0.252, *P* = 0.779; Bonferroni’s Multiple Comparison Test; *P* > 0.05 (Venus vs. ChETA), *P* > 0.05 (ChETA vs. eArchT), *P* > 0.05 (Venus vs. eArchT), Right: Venus, 15.3 ± 8.4 s, ChETA, 13.4 ± 4.2 s, eArchT, 8.0 ± 2.1 s; Kruskal−Wallis test; Kruskal−Wallis statistic = 0.281, *P* = 0.869; Dunn’s Multiple Comparison Test; *P* > 0.05 (Venus vs. ChETA, ChETA vs. eArchT, Venus vs. eArchT)). **c** One day after the open field test, time in open arms in the elevated plus maze test (300 s) was examined in the presence of light illumination to the DRN. Representative heat map analysis of one animal in each group (left). Data represent mean ± SEM. *n* = 11 (Venus), 13 (ChETA), 11 (eArchT) rats (right). *P < 0.05 vs. Venus. (Venus, 93.8 ± 21.0 s, ChETA, 68.6 ± 14.5 s, eArchT, 36.5 ± 8.8 s; Kruskal−Wallis test; Kruskal−Wallis statistic = 6.919, *P* = 0.032; Dunn’s Multiple Comparison Test; *P* > 0.05 (Venus vs. ChETA), *P* < 0.05 (Venus vs. eArchT), *P* > 0.05 (ChETA vs. eArchT))

To investigate the effect of DRN serotonergic neurons on mood-related behaviors also in rats, we performed the forced swim test, the open field test, and the elevated plus maze test (Fig. 4, Supplementary Figure S18). In the forced swim test, rats injected with srTPH2:ChETA showed decreased immobility compared to srTPH2:Venus-injected animals (Fig. 4a), while rats injected with srTPH2:eArchT had similar immobility to srTPH2:Venus-injected animals (Fig. 4a). In the open field test, there was no significant difference in the covered distance among srTPH2:Venus-, srTPH2:ChETA- and srTPH2:eArchT-injected rats (Fig. 4b). These results demonstrate that acute selective stimulation of the DRN serotonergic neurons induces antidepressant-like effect in rats. We observed no significant differences in exploration time in the center zone in the open field test among srTPH2:Venus-, srTPH2:ChETA- and srTPH2:eArchT-injected rats (Fig. 4b). However, in the elevated plus maze test, rats injected with srTPH2:eArchT spent significantly less time in the open arms than srTPH2:Venus-injected animals, while rats injected with srTPH2:ChETA had similar exploration time in the open-arms to srTPH2:Venus-injected animals (Fig. 4c). These results indicate that selective inhibition of the DRN serotonergic neurons has an anxiogenic effect in rats although its potency seems to depend on the paradigm.

**Fig. 5:**
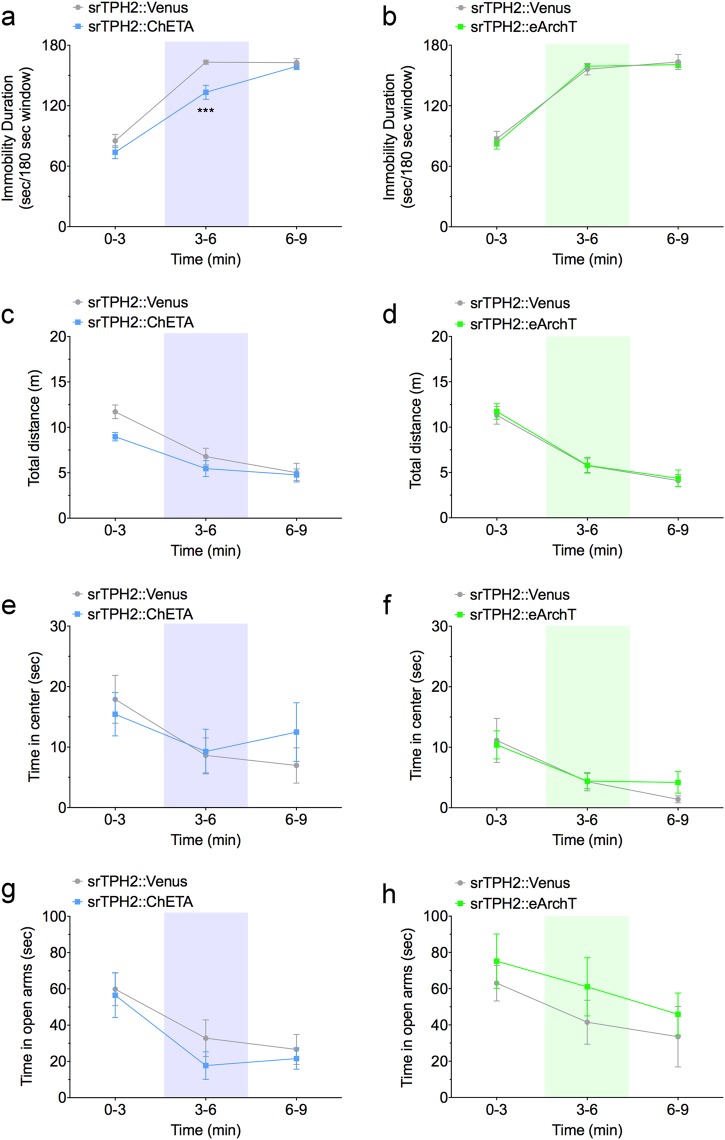
Effect of transient activation of rat DRN serotonergic neurons on stress-coping and anxiety-related behaviors. **a**, **b** One week after injection of srTPH2:ChETA, srTPH2:eArchT, or srTPH2:Venus in the DRN, immobility duration in the forced swim test was examined in the presence of light illumination to the DRN. Light was applied from 3 to 6 min of the test session. Data represent mean ± SEM of immobility duration in each time window of the test session. *n* = 10 (Venus in **a**), 11 (ChETA), 9 (Venus in **b**), 11 (eArchT) rats. ***P < 0.001 vs. Venus (**a** Venus 0−3, 85.2 ± 6.4 s, 3−6, 163.2 ± 1.9 s, 6−9, 162.7 ± 3.9 s, ChETA 0−3, 73.8 ± 6.3 s, 3−6, 133.3 ± 6.9 s, 6−9, 159.0 ± 3.1 s; two-way ANOVA; Interaction, *F*(2, 38) = 4.75, *P* = 0.015, Time, *F*(2, 38) = 200.1, *P* < 0.0001, Opto, *F*(1, 19) = 7.77, *P* = 0.012; Bonferroni posttests; 0−3, *P* > 0.05, 3−6, *P* < 0.001, 6−9, *P* > 0.05, **b** Venus 0−3, 87.3 ± 7.4 s, 3−6, 156.2 ± 5.6 s, 6−9, 163.4 ± 7.4 s, eArchT 0−3, 83.2 ± 6.0 s, 3−6, 159.1 ± 2.7 s, 6−9, 160.7 ± 2.9 s; two-way ANOVA; Interaction, *F*(2, 36) = 0.367, *P* = 0.695, Time, *F*(2, 36) = 198, *P* < 0.0001, Opto, *F*(1, 18) = 0.048, *P* = 0.830; Bonferroni posttests; 0−3, *P* > 0.05, 3−6, *P* > 0.05, 6−9, *P* > 0.05). **c−f** One week after injection of srTPH2:ChETA, srTPH2:eArchT, or srTPH2:Venus in the DRN, open field test was performed in the presence of light illumination to the DRN. Light was applied from 3 to 6 min of the test session. Data represent mean ± SEM of traveled distance or spent time in the center zone in each time window of the test session. *n* = 10 (Venus in **c**, **e**), 11 (ChETA), 9 (Venus in **d**, **f**), 11 (eArchT) rats. No significant difference was observed (**c** Venus 0−3, 11.7 ± 0.7 m, 3−6, 6.8 ± 0.9 m, 6−9, 5.0 ± 1.0 m, ChETA 0−3, 9.0 ± 0.5 m, 3−6, 5.5 ± 0.9 m, 6−9, 4.8 ± 0.6 m; two-way ANOVA; Interaction, *F*(2, 38) = 2.14, *P* = 0.132, Time, *F*(2, 38) = 44.34, *P* < 0.0001, Opto, *F*(1, 19) = 2.68, *P* = 0.118; Bonferroni posttests; 0−3, *P* > 0.05, 3−6, *P* > 0.05, 6−9, *P* > 0.05, **d** Venus 0−3, 11.3 ± 1.0 m, 3−6, 5.8 ± 0.8 m, 6−9, 4.1 ± 0.7 m, eArchT 0−3, 11.7 ± 0.9 m, 3−6, 5.8 ± 0.9 m, 6−9, 4.4 ± 0.9 m; two-way ANOVA; Interaction, *F*(2, 36) = 0.023, *P* = 0.977, Time, *F*(2, 36) = 38.06, *P* < 0.0001, Opto, *F*(1, 18) = 0.119, *P* = 0.734; Bonferroni posttests; 0−3, *P* > 0.05, 3−6, *P* > 0.05, 6−9, *P* > 0.05, **e** Venus 0−3, 17.9 ± 3.9 s, 3−6, 8.6 ± 2.9 s, 6−9, 7.0 ± 2.9 s, ChETA 0−3, 15.4 ± 3.6 s, 3−6, 9.3 ± 3.7 s, 6−9, 12.5 ± 4.8 s; two-way ANOVA; Interaction, *F*(2, 38) = 1.12, *P* = 0.338, Time, *F*(2, 38) = 4.98, *P* = 0.012, Opto, *F*(1, 19) = 0.082, *P* = 0.778; Bonferroni posttests; 0−3, *P* > 0.05, 3−6, *P* > 0.05, 6−9, *P* > 0.05, **f** Venus 0−3, 11.1 ± 3.6 s, 3−6, 4.3 ± 1.5 s, 6−9, 1.4 ± 0.6 s, eArchT 0−3, 10.4 ± 2.3 s, 3−6, 4.4 ± 1.3 s, 6−9, 4.2 ± 1.8 s; two-way ANOVA; Interaction, *F*(2, 36) = 0.381, *P* = 0.686, Time, *F*(2, 36) = 7.893, *P* = 0.001, Opto, *F*(1, 18) = 0.211, *P* = 0.652; Bonferroni posttests; 0−3, *P* > 0.05, 3−6, *P* > 0.05, 6−9, *P* > 0.05). **g**, **h** One week after injection of srTPH2:ChETA, srTPH2:eArchT, or srTPH2:Venus in the DRN, elevated plus maze test was performed in the presence of light illumination to the DRN. Light was applied from 3 to 6 min of the test session. Data represent mean ± SEM of spent time in the open arms in each time window of the test session. *n* = 10 (Venus in **g**), 11 (ChETA), 9 (Venus in **h**), 11 (eArchT) rats. No significant difference was observed (**g** Venus 0−3, 59.9 ± 9.1 s, 3−6, 32.8 ± 10.1 s, 6−9, 26.6 ± 8.3 s, ChETA 0−3, 56.5 ± 12.3 s, 3−6, 17.7 ± 7.6 s, 6−9, 21.5 ± 5.9 s; two-way ANOVA; Interaction, *F*(2, 38) = 0.271, *P* = 0.764, Time, *F*(2, 38) = 10.21, *P* = 0.0003, Opto, *F*(1, 19) = 0.900, *P* = 0.355; Bonferroni posttests; 0−3, *P* > 0.05, 3−6, *P* > 0.05, 6−9, *P* > 0.05, **h** Venus 0−3, 63.1 ± 9.8 s, 3−6, 41.5 ± 12.1 s, 6−9, 33.5 ± 16.7 s, eArchT 0−3, 75.2 ± 15.0 s, 3−6, 61.0 ± 16.1 s, 6−9, 45.9 ± 11.7 s; two-way ANOVA; Interaction, *F*(2, 36) = 0.111, *P* = 0.895, Time, *F*(2, 36) = 5.59, *P* = 0.008, Opto, *F*(1, 18) = 0.741, *P* = 0.401; Bonferroni posttests; 0−3, *P* > 0.05, 3−6, *P* > 0.05, 6−9, *P* > 0.05)

To investigate whether promotion of active coping in rats by DRN serotonergic neurons is time-locked, we examined the effect of transient optogenetic stimulation/inhibition in rats. Consistent to the result in mice, optogenetic stimulation of the rat DRN serotonergic neurons significantly decreased the immobility duration in the forced swim test when blue light was applied (Fig. 5a), whereas inhibition of them did not significantly affect the immobility duration throughout the test session (Fig. 5b). We also performed the open field and elevated plus maze tests using transient light illumination in rats (Fig. 5c−h, Supplementary Figures S19, S20). There were no significant differences in total distance traveled (Fig. 5c, d) and time in center zone (Fig. 5e, f) in the open field test, and time in open arms (Fig. 5g, h) in the elevated plus maze test. Furthermore, we examined the effect of optogenetic activation and inhibition of rat DRN serotonergic neurons in light−dark transition test. However, there was no significant difference in the time spent in the dark area (Supplementary Figure S13c, d). Moreover, we performed real-time place preference test to investigate the effect with optogenetic activation and inhibition on valence. Consistent with the results in mice, there was no significant difference in the spent time in the area associated with light stimulation (Supplementary Figure S14c, d, S21, S22). These results indicate that stimulation of DRN serotonergic neurons rapidly promotes active coping to stress.

## Discussion

In the present study, we have developed the new generation of the LVV capable of specific manipulation of serotonergic neurons in rats and mice which are sufficiently potent and selective to be used to express optogenetic actuators. To our knowledge, this is the first report investigating the behavioral consequences of specific manipulation of rat serotonergic neurons in vivo, although transgenic mice have been successfully used for this purpose [26, 28]. Multiple lines of evidence have demonstrated that serotonin is involved in mood regulation, social interaction, cognition, impulsivity, aggression, and decision making [14, 16, 27, 40, 41]. Although importance of these brain functions is obvious, sufficiency and necessity of serotonergic neuronal activity for manifestation of these functions are rarely demonstrated in rats and higher animals. In this context, our vectors pave the way to compare the impact of serotonergic neuronal activity between species. Specifically, considering differences in the efficacy of the mouse and rat TPH2 promoters in this study, using primate orthologs of the rodent sequences used here might be useful for achieving specific targeting of serotonergic neurons in non-human primates which can perform far more complicated tasks than rodents [42]. Previously, Warden et al. have demonstrated that specific activation of excitatory inputs from the medial prefrontal cortex to the DRN elicits time-locked increase in struggling behavior in rat forced swim test paradigm [43]. In contrast, Challis et al. clearly demonstrated that specific inhibition of these neurons elicits antidepressant-like effect in a social defeat paradigm [44]. Therefore, it is still controversial whether the stimulation of this pathway has antidepressive or prodepressive effect. Although Warden et al. showed that nonselective activation of DRN neurons increases struggling behavior, it also induces time-locked hyperlocomotion in the open field test, indicating that this struggling behavior may result from hyperlocomotion, rather than represent an antidepressant-like effect [43]. In the present study, we demonstrate that acute specific stimulation of the DRN serotonergic neurons is sufficient for inducing antidepressant-like effect in mice and rats by using these LVVs. It should be noted that this effect was observed only in the second half of the session in mice while SSRI was effective throughout the session. Considering that acute treatment with an SSRI dose-dependently decreased the immobility duration throughout the test session, it is unlikely that insufficient activation of the DRN serotonergic neurons can explain the difference between the optogenetic stimulation and SSRI treatment. One possible explanation is that serotonergic neurons in several nuclei mediate the effect of SSRIs, with DRN being only one of them. Further analysis is necessary for the dissection of neural circuits mediating the effect of antidepressants. Additionally, we found that antidepressant-like effect induced by activation of DRN serotonergic neurons disappeared when illumination was turned off. These results highlight the importance of activity of DRN serotonergic neurons for stress-coping. On the other hand, they also suggest that acute activation of serotonergic neurons is not sufficient for long-term antidepressant-like effect. Therefore, therapeutics inducing long-term activation of serotonergic neurons may be effective for long-term active coping with stress. In addition to the antidepressant-like effect, we found that optogenetic inhibition of rat DRN serotonergic neurons increased anxiety-related behaviors in the elevated plus maze test but not in the open field test. The elevated plus maze test utilizes unconditioned fear of heights and/or open spaces, while the open field test relies upon that of open spaces. The apparent discrepancy may be partly explained by different patterns of neural activity between two tests, which we attempted to suppress optogenetically. In mice, we did not observe effect of optogenetic inhibition of DRN serotonergic neurons on anxiety-related behaviors. Gutknecht et al. demonstrated that genetic deletion of mouse TPH2 reduced the anxiety-related behaviors in the elevated plus maze test when chronic mild stress was applied [45]. Therefore, different level of baseline stress may affect the relative contribution of serotonergic neurons on anxiety-related behaviors, which might underlie the apparent discrepancy among species. Moreover, all behavioral analyses in this study were performed in the light cycle of the day. Considering that elevated plus maze test experiments in the dark cycle of day results in a shorter open arm time [46], it may be possible that the DRN activity impacts behavior in the elevated plus maze test differently during light and dark phases of the day. Another possible explanation for this discrepancy is insufficient optogenetic inhibition in mice in vivo, although we have confirmed that inhibition is produced by green light illumination ex vivo. Furthermore, immunohistochemical quantification of infected DRN serotonergic neurons (Supplementary Figure S4, S5) indicates that a greater proportion of DRN serotonergic neurons are infected by these LVV in mice than in rats, in which optogenetic inhibition affected anxiety-related behavior. For this reason we do not think that the inhibition in vivo was insufficient, although further electrophysiological analysis in vivo is needed. Another possible explanation is that effect of serotonergic signaling on anxiety differs between species. Indeed, acute administration of 8-OH-DPAT, a 5HT_1A_ agonist, induces anxiolytic effect in rats but not in mice without sedation [47, 48], supporting this possibility. Marcinkiewcz et al. demonstrated that stimulation of mouse serotonergic neurons projected from the DRN to the bed nucleus of stria terminalis (BNST) is anxiogenic [26], while Ohmura et al. [28] and our data show that stimulation of DRN serotonergic neurons does not affect the level of anxiety. Because the DRN serotonergic neurons send projections to a number of brain areas, it is possible that some of them encode anxiolytic signals, while others, including BNST-projecting cells, are anxiogenic. Effects caused by the inhibition of neurons should not be observed in the absence of their activity. Thus, it is possible that anxiolytic serotonergic projections in mice is not activated in the elevated plus maze test but those in rats is activated, resulting in the apparently different sensitivity to optogenetic manipulation between species, although further analysis of serotonergic activity using fiberphotometry [49] or miniaturized fluorescence microscopy [50] within these behavioral paradigms would be required to further investigate this hypothesis.

In summary, our data provide direct evidence that selective stimulation of serotonergic neurons in the DRN is sufficient to induce a time-locked antidepressant-like effect in mice and rats. Furthermore, we show that optogenetic inhibition of the DRN serotonergic neurons increased anxiety-related behaviors in rats, indicating that in this species DRN serotonergic neurons contribute to the control of anxiety.

## Funding and disclosure

This work was partly supported by Grant- in-Aid for Scientific Research from JSPS (to K.N., H.S., T.N., H.H., and S. Kaneko), AMED (to H.H.), MRC (MR/L020661/1; to S. Kasparov), BBSRC (BB/L019396/1; to S. Kasparov), and Research Grant from Research Foundation for Opto-Science and Technology, from Nakatani Foundation for advancement of measuring technologies in biomedical engineering, and from Takeda Science Foundation (to K.N.). The authors declare no competing interests.

## Electronic supplementary material

APC Form

Supplementary Information

Supplementary Information
