# Supplementary material for: Manipulation of dorsal raphe serotonergic neurons modulates active coping to inescapable stress and anxiety-related behaviors in mice and rats
Source: Neuropsychopharmacology. 2018 Oct 30;44(4):721–32. doi: 10.1038/s41386-018-0254-y (PMC6372597; doi:10.1038/s41386-018-0254-y)
Supplement: Supplementary file 2 — Supplementary Information [file 41386_2018_254_MOESM2_ESM.pdf]

Supplementary Materials and Methods

Production and purification of self-inactivating lentiviral vector (LVV). Lenti-X 293T cells (Clontech, Mountain View, CA, USA) were grown to 60-70% confluency, and packaging vectors and shuttle construct were transfected with polyethylenimine (Polysciences, Warrington, PA, USA). After 16-18 hours of incubation, supernatant was harvested, and fresh media was added to the culture. After 30 hours of incubation, supernatant was collected and mixed with the first harvest. The supernatants were filtrated through the 0.45 μm pore size PVDF membrane (Millex-HV, Merck Millipore, Billerica, MA, USA) and ultracentrifuged for 2 hours at 23,000 rpm in SW-28 rotor (Beckman-Coulter, Brea, CA, USA). The precipitate was resuspended in PBS and stored at -80°C. The titer of LVV was measured by p24 ELISA (BioAcademia, Osaka, Japan) and estimated to be about 4-5×10^{10} IU/mL.

Stereotaxic surgeries.

Stereotaxic surgeries were conducted using a small animal stereotaxic frame (Narishige, Tokyo, Japan) according to the Brain Atlas (Franklin and Paxinos, 2007; Paxinos and Watson, 2007). The adult animals were anesthetized with sodium pentobarbital (50 mg/kg, i.p., Kyoritsu Seiyaku, Tokyo, Japan) and rat pups were anesthetized with 3% isofluran (Escain, Pfizer, Tokyo, Japan). The lentiviral vectors (LVV) were microinjected at 1 μL for mice and rat pups or 5 μL for adult rats into the DRN (mouse: AP -4.4 mm, ML 0 mm, DV +3.4 mm from bregma, rat: AP -7.7 mm, ML 0 mm, DV +6.5 mm from bregma, rat pups: AP +1.4 mm, ML 0 mm, DV +6.0 mm from lambda).

We used LVV encoding Venus (Nagai *et al*, 2002) (TPH2::Venus), a ChR2 variant, ChETA (Gunaydin *et al*, 2010), fused with eYFP (TPH2::ChETA), or a light-activated proton pump, eArchT (Mattis *et al*, 2011) fused with eYFP (TPH2::eArchT) under the control of the TPH2 promotor. For behavioral experiments, 1-2 weeks after the viral injection the animals were implanted with a fiber optic cannula so that the tip of the cannula was placed just above the dorsal border of the DRN. The experimenters which carried out behavioral analyses were blinded to the group allocation. After behavioral analyses, all mice and rats were sacrificed, and the LVV infection was verified immunohistochemically. The data points obtained from the mice or rats with failed LVV infection were excluded. The numbers of the animals used were determined so that sufficient statistical power could be reached, according to the previous reports (Tye *et al*, 2012).

Histology. The animals were deeply anesthetized with pentobarbital and transcardially perfused with PBS followed by 4% paraformaldehyde (Nacalai Tesque, Kyoto, Japan) in PBS. After perfusion fixation, the brains were removed, equilibrated in 15% sucrose in PBS for overnight and frozen using dry ice. The brains were cryosectioned into 30 µm-thick coronal sections with the cryostat (Leica CM3050S; Leica Biosystems, Nussloch, Germany) and stored at -80°C until immunohistochemical processing.

For immunohistochemistry, the sections were immersed in 0.25% Triton-X 100 (Nacalai Tesque) for permeabilization and then incubated overnight at 4°C with rabbit polyclonal anti-green fluorescent protein (GFP) antibody (1:2000; A-11122, Life Technologies,

Carlsbad, CA, USA), rabbit monoclonal anti-c-fos antibody (1:1000; #2250, Cell Signaling Technology, Danvers, MA, USA) and sheep polyclonal anti-tryptophan hydroxylase (TPH) antibody (1:200; AB1541, Merck Millipore), followed by incubation with Alexa Fluor 488- or 594-labeled donkey anti-rabbit IgG (1:200; Life Technologies) and Alexa Fluor 647-labeled donkey anti-sheep IgG (1:200; Life Technologies) for 2 hours at room temperature. The sections were then washed in PBS and mounted on glass with Vectashield (Vector Laboratories, Burlingame, CA, USA). Immunofluorescence was visualized using a laser scanning confocal microscopy (Fluoview FV10i, Olympus, Tokyo, Japan).

Electrophysiology. After at least one week recovery period, the animals injected with the LVV were deeply anesthetized by isoflurane and decapitated. The brain was collected in ice-cold cutting solution (120 mM NMDG-Cl, 2.5 mM KCl, 26 mM $NaHCO_3$, 1.25 mM NaH_2PO_4, 0.5 mM $CaCl_2$, 7 mM $MgCl_2$, 15 mM D-glucose, 1.3 mM ascorbic acid, pH 7.2). Coronal midbrain slices (200-μm thick) were prepared with a vibratome (VT1000S, Leica Biosystems), and recovered in oxygenated artificial cerebrospinal fluid (aCSF; 124 mM NaCl, 3 mM KCl, 26 mM $NaHCO_3$, 1 mM NaH_2PO_4, 2.4 mM $CaCl_2$, 1.2 mM $MgCl_2$, and 10 mM D-glucose, pH 7.3) at 32°C for at least 1 h before recording. After recovery, individual slices were transferred to a recording chamber with continuous perfusion of oxygenated aCSF. Individual neurons were visualized with an upright fluorescence microscope equipped with a 40× water-immersion objective lens (Carl Zeiss, Oberkochen, Germany) and a CCD camera.

The eYFP-positive neurons were used for recording.

Whole-cell recordings were performed with an EPC 9 amplifier (HEKA, Pfalz, Germany), and the data were recorded using Patchmaster software. The resistance of the electrodes was 4–7 MΩ when filled with the internal solution (140 mM K-gluconate, 5 mM KCl, 10 mM HEPES, 2 mM Na-ATP, 2 mM $MgCl_2$, 0.2 mM EGTA, pH 7.3 adjusted with KOH). All experiments were performed in the presence of CNQX (10 μM), AP-V (25 μM) and bicuculline (10 μM) to block AMPA, NMDA and $GABA_A$ receptors, respectively. For recording ChETA mediated current, tetrodotoxin (1 μM) was also perfused to block voltage-dependent Na^+-channels.

Vector Construction. For construction of rTPH2-Venus, Venus fragment was amplified by PCR from pCSII-Venus-PRE (generous gift from Drs. Miyoshi and Miyawaki (RIKEN, Japan)), digested with NotI/SpeI, and ligated into pTYF-G4BS-rTPH2-GluA1-IRES-Gal4p65 (rTPH2-GluA1) treated with NotI/SpeI. For construction of rTPH2-Venus-WPRE (srTPH2::Venus), rTPH2-GluA1-WPRE which was constructed through Infusion reaction (Clontech) with PCR-amplified IRES, Gal4p65, WPRE and SalI-treated rTPH2-GluA1, was digested with NotI/SpeI, and ligated with PCR-amplified Venus fragment digested with NotI/XbaI. For construction of rTPH2-ChETA-eYFP-WPRE and rTPH2-eArchT3.0-eYFP-WPRE, ChETA-eYFP and eArchT3.0-eYFP fragments were PCR amplified from pLenti-CaMKII-ChETA-eYFP and pLenti-CaMKII-eArchT3.0-eYFP (both obtained from Addgene), respectively. Amplified fragments were digested with AscI/SpeI, and

ligated into rTPH2-GluA1-WPRE treated with MluI/SpeI. For construction of mTPH2-Venus-WPRE (smTPH2::Venus), mouse TPH2 promoter was amplified from mouse genomic DNA, digested with SacII/MluI and ligated into rTPH2-Venus-WPRE digested with SacII/MluI. For construction of mTPH2-ChETA-eYFP-WPRE (smTPH2::ChETA) and mTPH2-eArchT3.0-eYFP-WPRE (smTPH2::eArchT), ChETA-eYFP and eArchT3.0-eYFP fragments were PCR amplified, digested with AscI/SpeI and ligated into mTPH2-GluA1-WPRE digested with MluI/SpeI. The constructs were verified by Sanger sequencing (Fasmac, Atsugi, Japan).

Behavioral tests

Light-dark transition test

The light-dark box arena consisted of two compartments divided into a light-zone (white acrylic cube, rats: 37 × 75 × 40 cm, mice: 25 × 50 × 50 cm, ~200 lux) and a dark-zone (white acrylic cube rats: 37 × 75 × 40 cm, mice: 25 × 50 × 50 cm, ~50 lux), with 10 cm width passage between them. Each animal was plugged into the fiber-optic patch cord, placed individually into the light-zone and permitted free exploration. The behavior of the animal was recorded with a camera over 9 min session; the recorded data were analyzed automatically using video tracking system (ANY-maze version 4.99, Stoelting, Wood Dale, IL, USA). Total time spent in the darkzone during a session was measured.

Social interaction test

Mice were acclimated to testing rooms under the red dim light for at least 30 min before the test. First, for habituation to a test environment, a fiber-optic patch cord was connetcted to the mouse which was kept for 2.5 min in an open field chamber (50 × 50 × 50 cm) with an empty wire mesh cage (10 × 6.5 cm) located at one end of the field. Consecutively, the same mouse was kept for 9 min in the same open field chamber with an unfamiliar C57BL/6J mouse enclosed in the wire mesh cage. Mouse behaviors were video monitored, and the trajectory of mouse ambulation was determined and recorded by ANY-MAZE (Stoelting Co., Wood Dale, IL, USA). The area surrounding the wire mesh cage (14 × 24 cm) defined as the interaction zone. Time in the social interaction zone during a session was measured.

Real-time place preference test

The real-time place preference arena consisted of a white acrylic cube (mice: 50 × 50 × 50 cm) which was divided into four zones of the same size (mice: 25 × 25 × 50 cm). One zone was illuminated when the animals entered it. Each animal was connected to the fiber-optic patch cord, placed individually into the arena and permitted free exploration. The behavior of the animal was recorded with a camera over a 15 min session for 3days; the recorded data were analyzed automatically using video tracking system. Time in the light-stimulated zone during a session was measured.

2-chamber real-time place preference test

The 2-chamber real-time place preference arena consisted of a pathway (10 × 20 × 30

cm) and two compartments in the same size (30 × 20 × 30 cm), and one of them was light-stimulated zone, where light illumination was delivered when the rat entered the zone. Each rat was connected to the fiber-optic patch cord, placed individually into the arena and permitted free exploration. The behavior of the rat was recorded with a camera over a 15 min session; the recorded data were analyzed automatically using video tracking system. Time in the light-stimulated zone during a session was measured.

Supplementary References

Franklin KBJ, Paxinos G (2007). *The Mouse Brain in Stereotaxic Coordinates Third Edition.* Academic Press: New York.

Nagai T, Ibata K, Park ES, Kubota M, Mikoshiba K, Miyawaki A (2002). A variant of yellow fluorescent protein with fast and efficient maturation for cell-biological applications. *Nat Biotechnol* **20**: 87–90.

Paxinos G, Watson C (2007) *The Rat Brain in Stereotaxic Coordinates Sixth Edition.* Academic Press: New York.

Tye KM, Mirzabekov JJ, Warden MR, Ferenczi EA, Tsai HC, Finkelstein J, *et al* (2012). Dopamine neurons modulate neural encoding and expression of depression-related behaviour. *Nature* **493**: 537–541.

Supplementary Figures

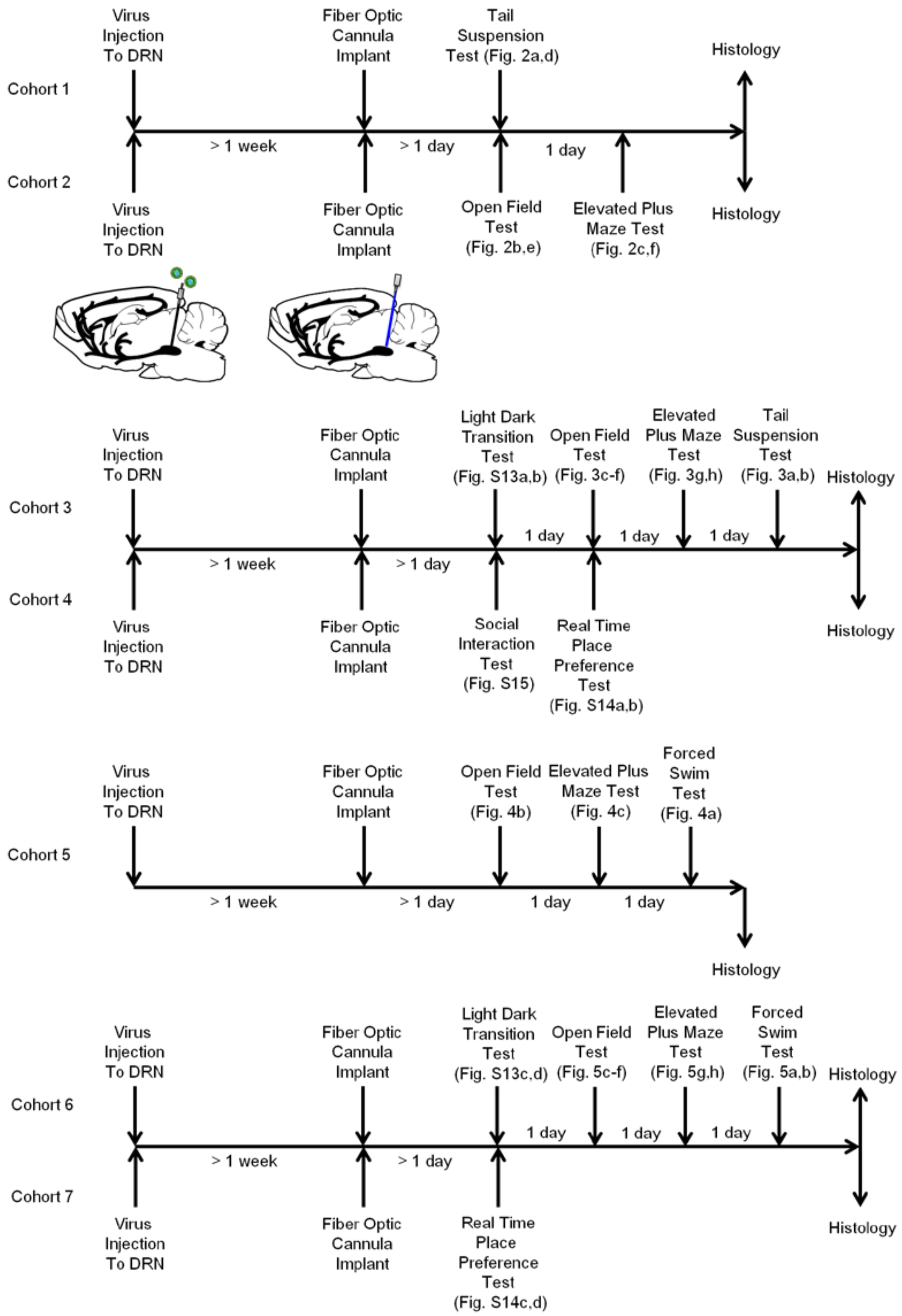

Supplementary Figure S1. Time course of behavioral experiments.

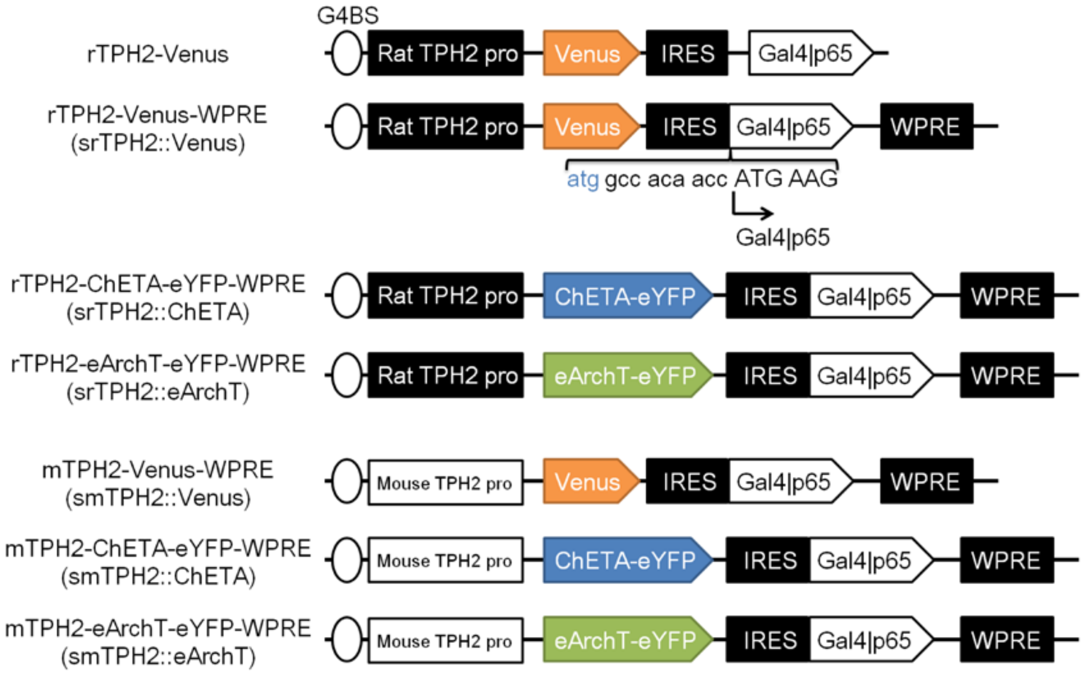

Supplementary Figure S2. Schematic of the LVV containing rat and mouse TPH2 promoters.

The construct rTPH2-Venus contains Gal4p65 and Gal4-binding sequence (G4BS) to increase transgene expression. To achieve stronger transgene expression, the sequence between IRES and GAl4p65 was deleted in srTPH2::Venus. In addition, WPRE was added to the downstream of Gal4p65. For optogenetic manipulation of the 5-HT neurons, Venus was replaced with ChETA-eYFP and eArchT-eYFP fragment, resulting in srTPH2::ChETA and srTPH2::eArchT, respectively. To apply these LVVs to mice, rat TPH2 promoter was replaced with its mouse homologue, resulting in smTPH2::Venus, smTPH2::ChETA and smTPH2::eArchT.

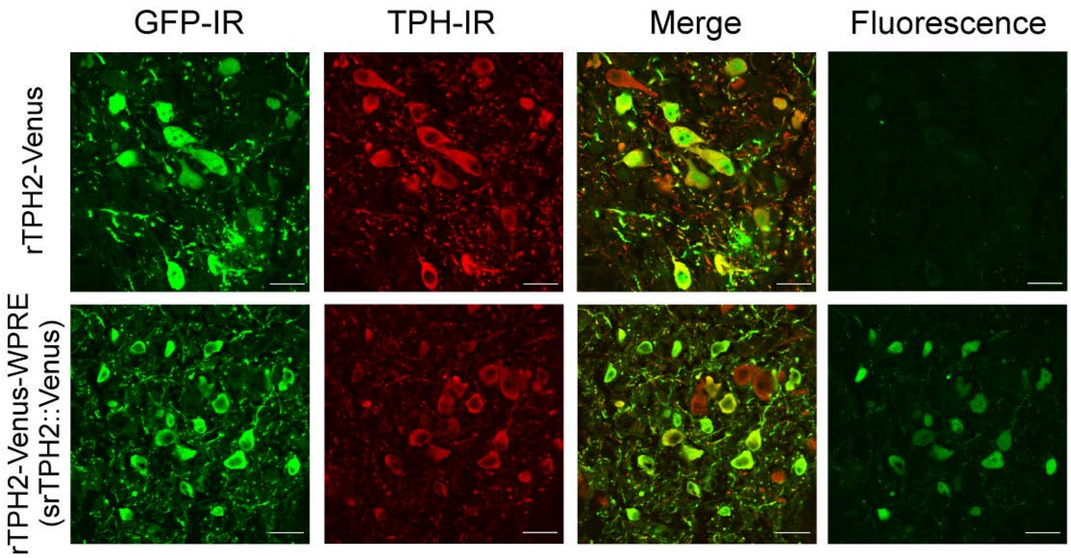

Supplementary Figure S3. Increased expression with srTPH2::Venus.

Rats were stereotaxically injected with rTPH2-Venus or rTPH2-Venus-WPRE (srTPH2::Venus) in the DRN. One week after injection, coronal sections containing the DRN were prepared and stained by anti-GFP and anti-TPH antibodies. After staining the sections were observed by confocal microscopy. Representative images from at least 3 rats were shown. Scale bars = 20 μm.

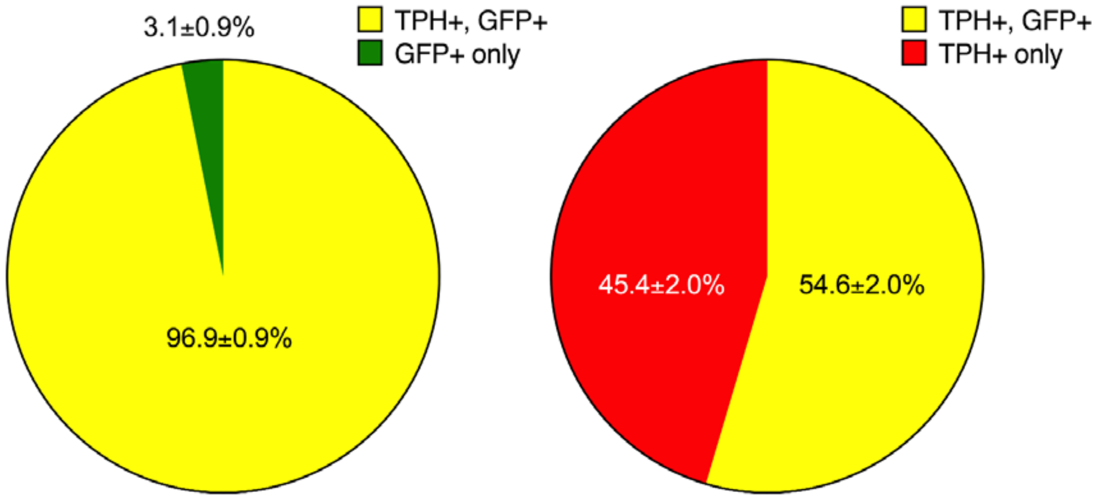

Supplementary Figure S4. Specificity of srTPH2::Venus in the rat DRN.

Rats were stereotaxically injected with srTPH2::Venus in the DRN. One week after injection, coronal sections containing the DRN were prepared and stained by anti-GFP and anti-TPH antibodies. After staining the sections were observed by confocal microscopy. The number of TPH- and GFP-immunoreactive cells were manually counted. Data represents means ± SEM of the percentage of TPH and GFP double-positive cells in GFP positive cells (left) or in TPH positive cells (right) are shown. n = 3 rats.

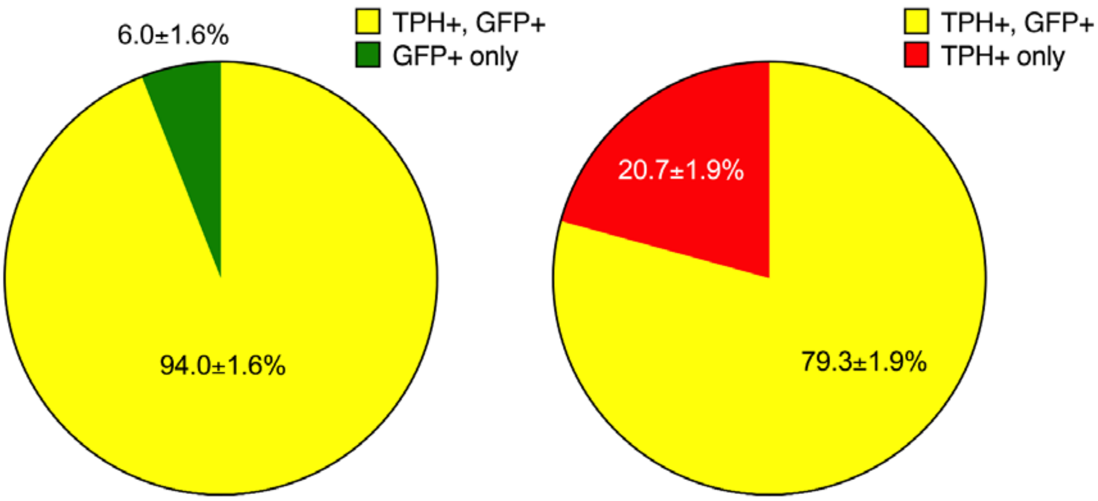

Supplementary Figure S5. Specificity of smTPH2::Venus in the mouse DRN.

Mice were stereotaxically injected with smTPH2::Venus in the DRN. One week after injection, coronal sections containing the DRN were prepared and stained by anti-GFP and anti-TPH antibodies. After staining the sections were observed by confocal microscopy. The number of TPH- and GFP-immunoreactive cells were manually counted. Data represents means ± SEM of the percentage of TPH and GFP double-positive cells in GFP positive cells (left) or in TPH positive cells (right) are shown. n = 3 mice.

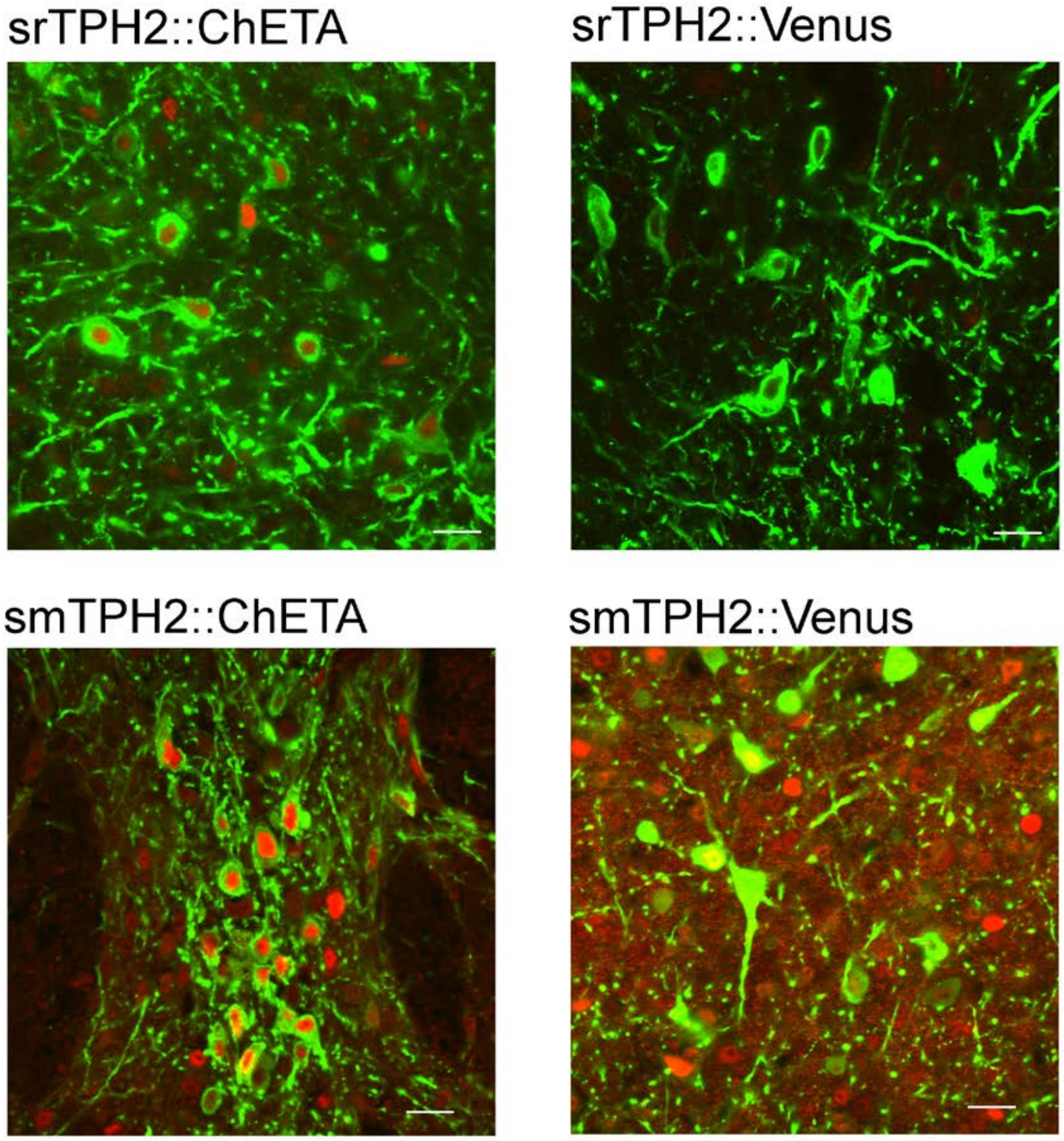

Supplementary Figure S6. Optogenetic activation induced c-fos immunoreactivity.

After infection of the DRN with srTPH2::ChETA, srTPH2::Venus, smTPH2::ChETA or smTPH2::Venus, blue light (20 Hz) was applied to the DRN for 3 min. Then animals were sacrificed, and the brain was collected, frozen and sectioned. Expression of transgenes (green) and c-fos (red) was investigated immunohistochemically. Scale bar = 20 μm.

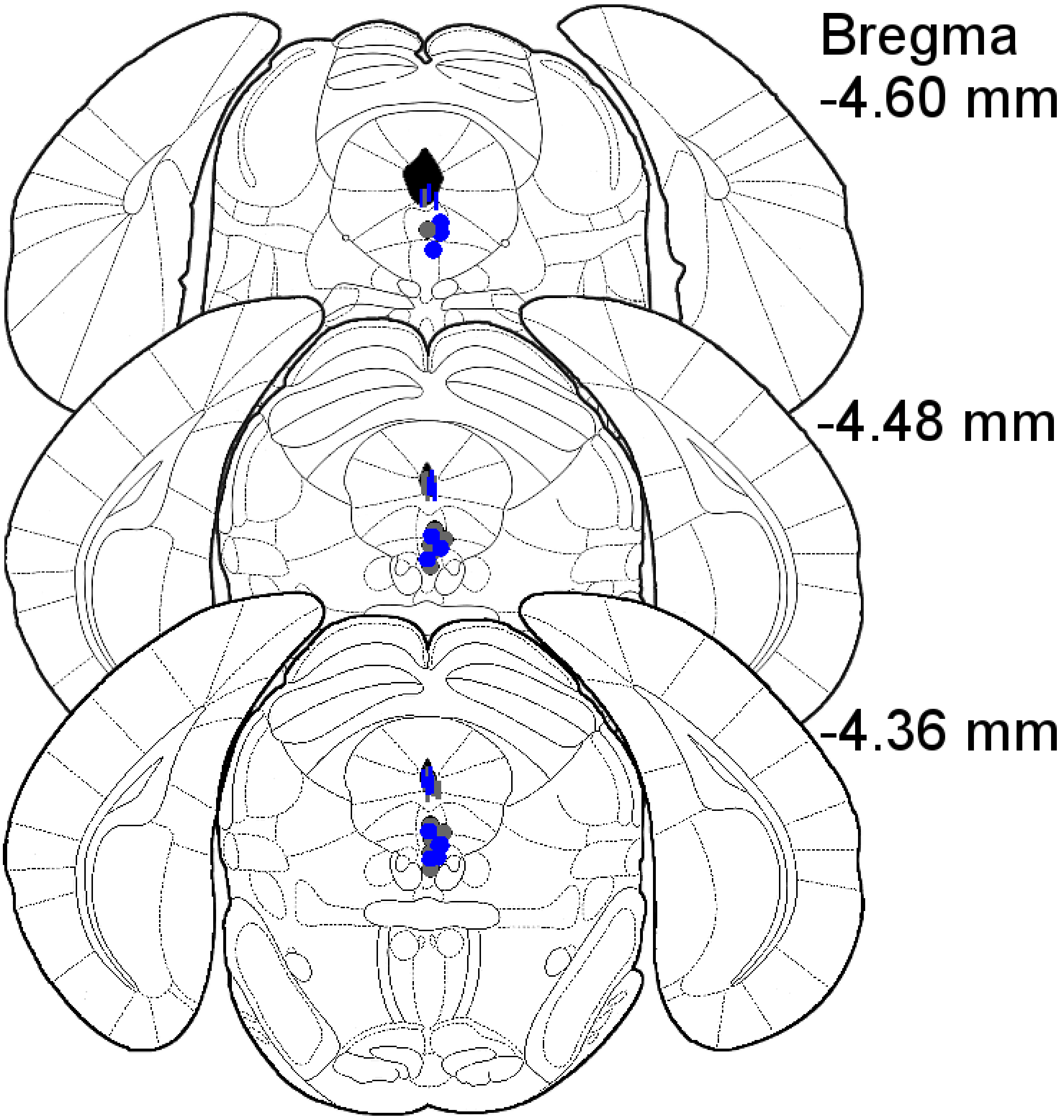

Supplementary Figure S7. Verification of LVV injection site (related to Figure 2).

After the completion of behavioral analysis, mice injected with smTPH2::Venus or smTPH2::ChETA were sacrificed, and the brain was collected, frozen and sectioned. The LVV injection site was histologically verified under the microscope. Gray circle: smTPH2::Venus, Blue circle: smTPH2::ChETA, Gray line: tip of fiber in smTPH2::Venus, Blue line: tip of fiber in smTPH2::ChETA.

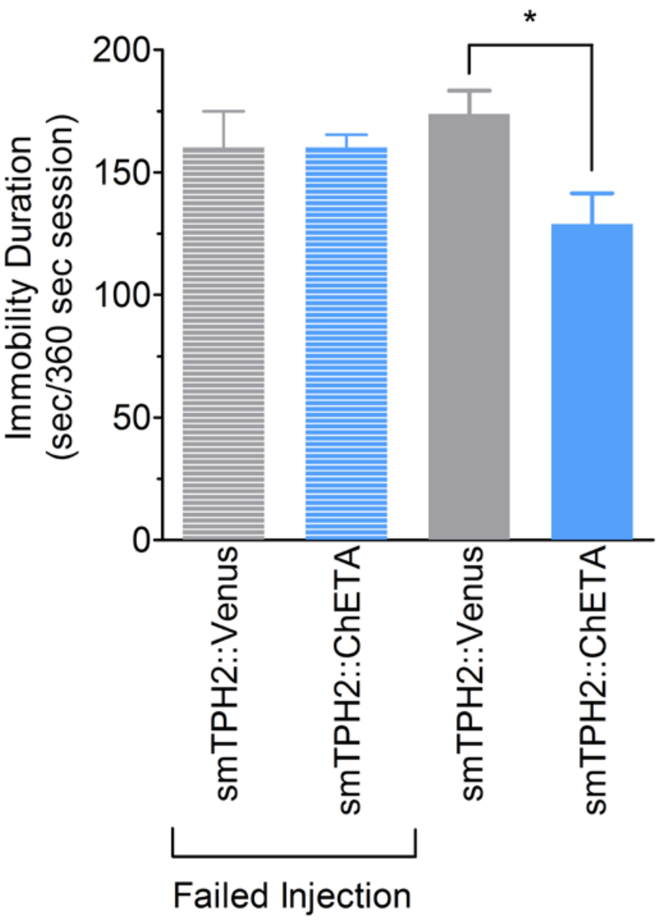

Supplementary Figure S8. Blue light illumination of the DRN in the mice with failed LVV injection did not induce antidepressant-like effect in the TST.

One week after injection of smTPH2::ChETA or smTPH2::Venus in the DRN, immobility duration in the TST was examined in the presence of blue light illumination to the DRN. Blue light was applied at 20 Hz with 0.5 ms duration from 15 min before the test to the end of the test session. Bar graphs show means ± SEM of immobility duration throughout the test session. n = 5 (Venus-failed), 6 (ChETA-failed), 12 (Venus), 10 (ChETA) mice. $^*P < 0.05$. (Venus-failed, 160.2 ± 14.7 sec, ChETA-failed, 160.2 ± 5.2 sec, Venus, 173.8 ± 9.5 sec, ChETA, 128.9 ± 12.6 sec; one-way ANOVA; $F(3, 29) = 3.492$, $P < 0.05$; Bonferroni's Multiple Comparison Test; $P < 0.05$ (Venus vs. ChETA), P

> 0.05 (other pairs))

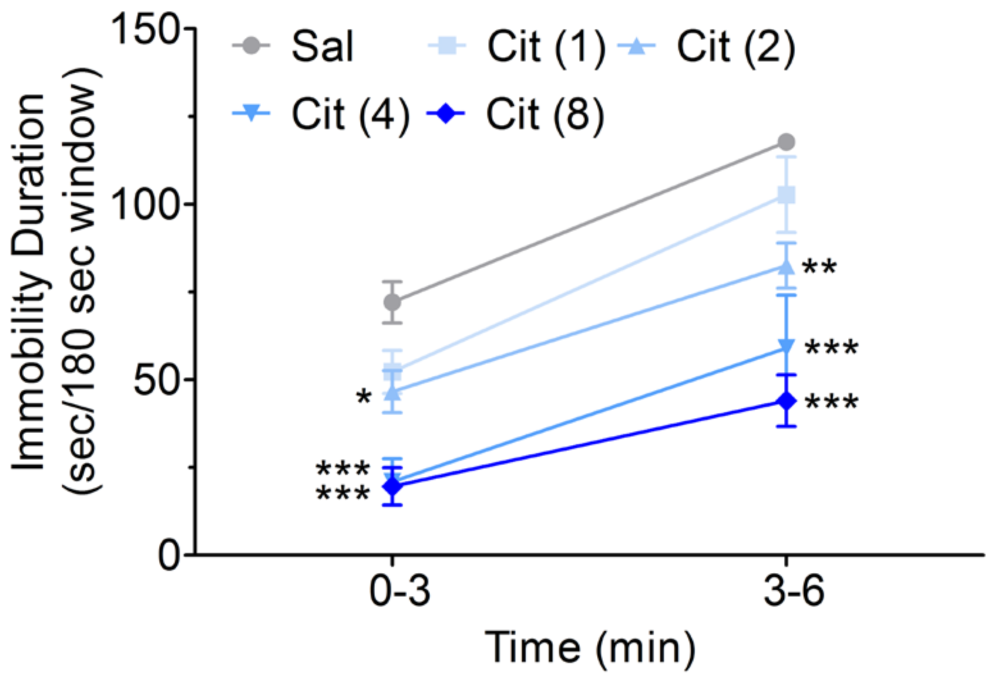

Supplementary Figure S9. Effect of an SSRI, citalopram, on immobility duration in the tail suspension test.

Mice were administered with saline (Sal) or citalopram (Cit: 1, 2, 4, 8 mg/kg, i.p.). Immobility duration in the tail suspension test was examined 30 min after drug administration. Data represents means ± SEM of immobility duration in first and second half of the test session. n = 8 (Sal, Cit (1, 2, 4)), 7 (Cit (8)) mice. *P < 0.05, **P < 0.01, ***P < 0.001 vs. Sal. (Sal, 72.1 ± 5.9 sec (0-3), 117.8 ± 1.5 sec (3-6), Cit1, 52.3 ± 6.1 sec (0-3), 102.7 ± 10.8 sec (3-6), Cit2, 46.6 ± 6.0 sec (0-3), 82.6 ± 6.4 sec (3-6), Cit4, 20.9 ± 6.6 sec (0-3), 59.1 ± 15.1 sec (3-6), Cit8, 19.7 ± 5.3 sec (0-3), 44.0 ± 7.3 sec (3-6); two-way ANOVA; F(4, 34) = 0.83, P = 0.516 (Interaction), F(4,34) = 19.41, P < 0.0001 (Drug), F(1, 34) = 65.46, P < 0.0001 (Time); Bonferroni posttest; Sal. vs. Cit1, 0-3, P > 0.05, 3-6, P > 0.05, Sal. vs. Cit2, 0-3, P < 0.05, 3-6, P < 0.01, Sal. vs. Cit4, 0-3, P < 0.001, 3-6, P < 0.001, Sal. vs. Cit8, 0-3, P < 0.001, 3-6, P < 0.001)

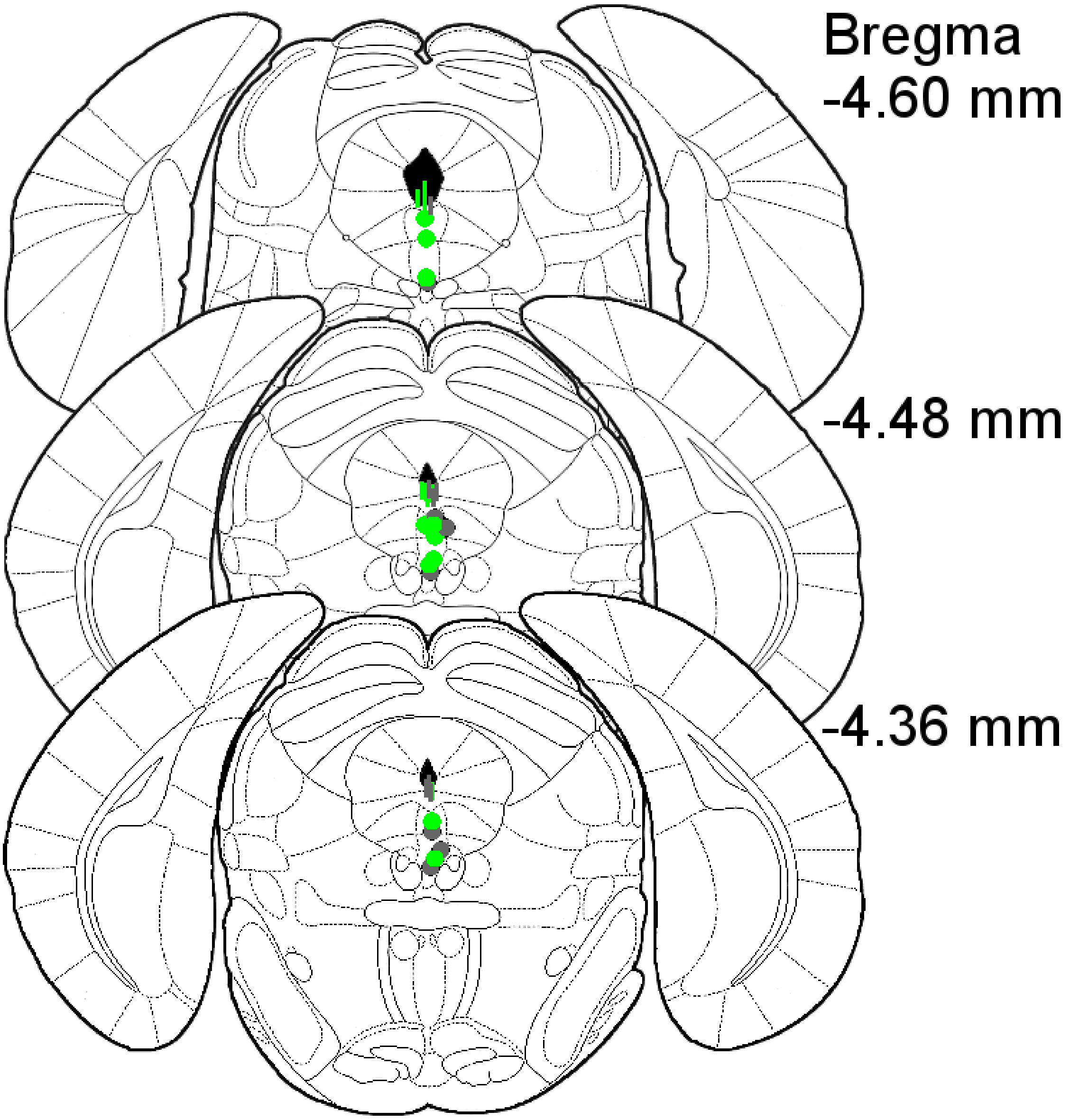

Supplementary Figure S10. Verification of LVV injection site (related to Figure 2).

After the completion of behavioral analysis, mice injected with smTPH2::Venus or smTPH2::eArchT were sacrificed, and the brain was collected, frozen and sectioned. The LVV injection site was histologically verified under the microscope. Gray circle: smTPH2::Venus, Green circle: smTPH2::eArchT, Gray line: tip of fiber in smTPH2::Venus, Green line: tip of fiber in smTPH2::eArchT.

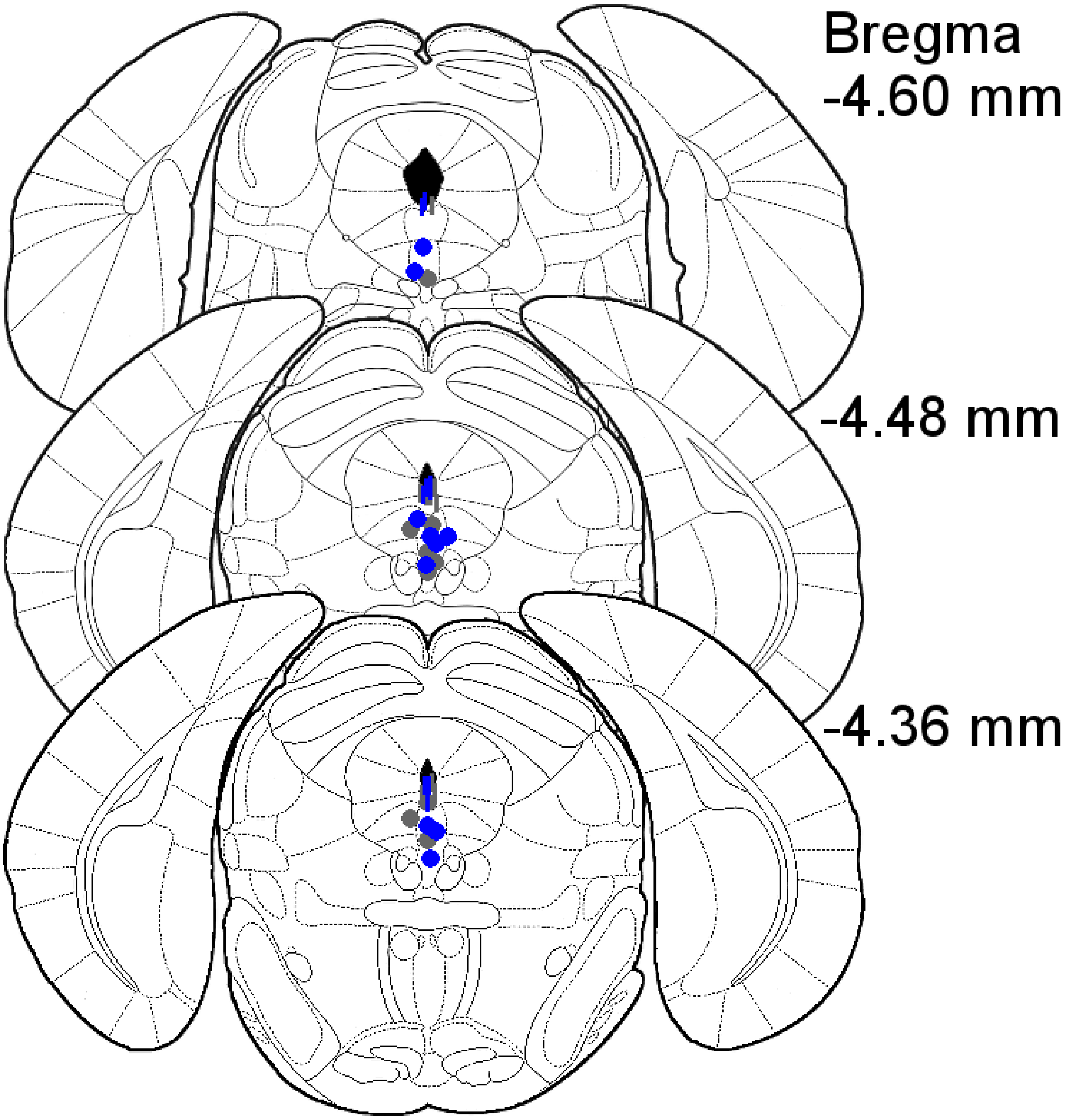

Supplementary Figure S11. Verification of LVV injection site (related to Figure 3).

After the completion of behavioral analysis, mice injected with smTPH2::Venus or smTPH2::ChETA were sacrificed, and the brain was collected, frozen and sectioned. The LVV injection site was histologically verified under the microscope. Gray circle: smTPH2::Venus, Blue circle: smTPH2::ChETA, Gray line: tip of fiber in smTPH2::Venus, Blue line: tip of fiber in smTPH2::ChETA.

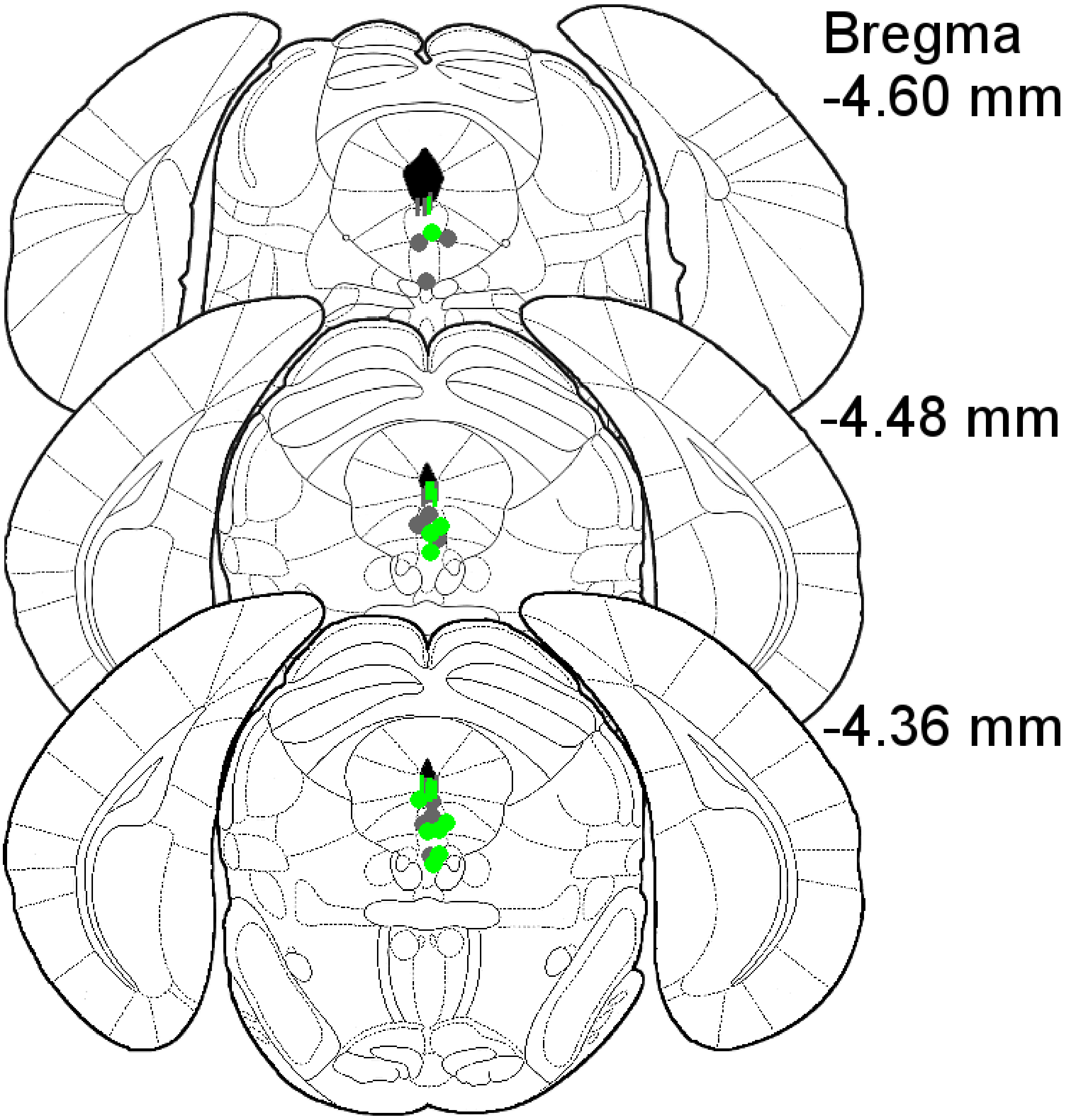

Supplementary Figure S12. Verification of LVV injection site (related to Figure 3).

After the completion of behavioral analysis, mice injected with smTPH2::Venus or smTPH2::eArchT were sacrificed, and the brain was collected, frozen and sectioned. The LVV injection site was histologically verified under the microscope. Gray circle: smTPH2::Venus, Green circle: smTPH2::eArchT, Gray line: tip of fiber in smTPH2::Venus, Green line: tip of fiber in smTPH2::eArchT.

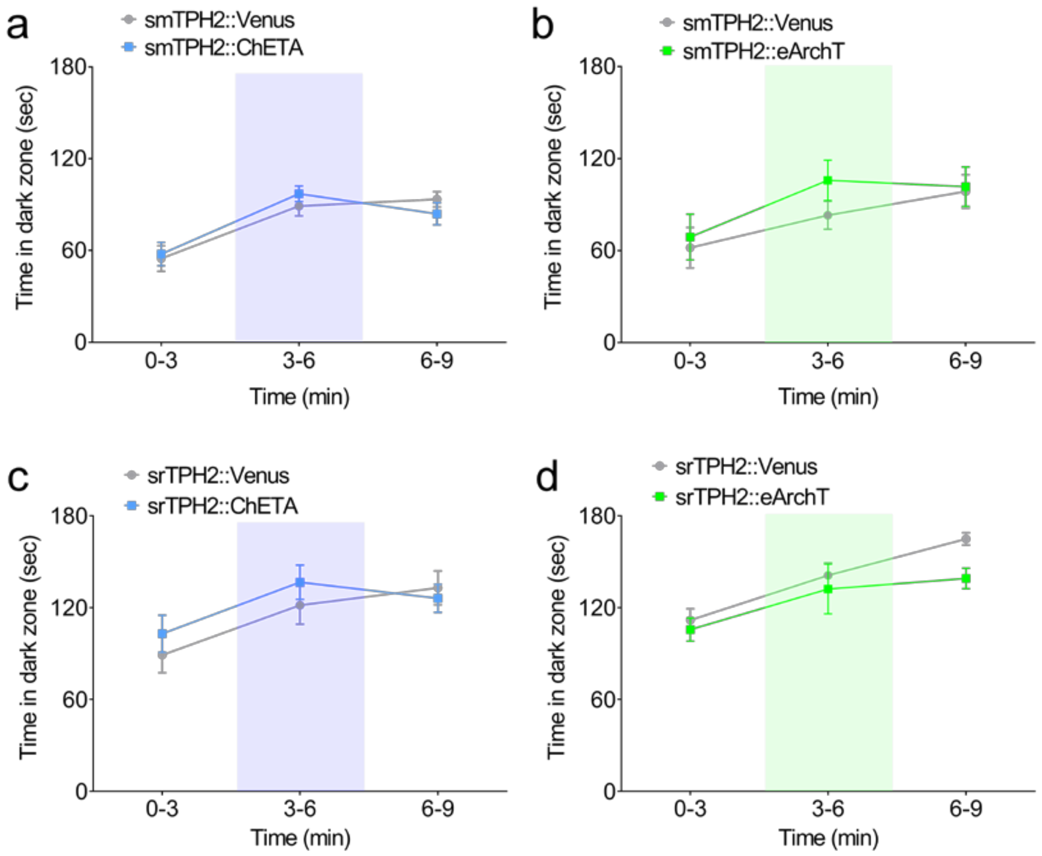

Supplementary Figure S13. Effect of optogenetic activation and inhibition in the light-dark transition test.

One week after injection of smTPH2::ChETA, smTPH2::eArchT, smTPH2::Venus, srTPH2::ChETA, srTPH2::eArchT, or srTPH2::Venus in the DRN, light-dark transition test was performed. Blue or green laser stimulation was applied from 3 to 6 min in the test session. Means ± SEM of spent time in the dark zone are shown. No significant difference was observed between ChETA- or eArchT-expressing animals and Venus controls. n = 9 (Venus, in a), 10 (ChETA, in a), 11 (Venus, in b), 10 (eArchT, in b), 10 (Venus, in c), 13 (ChETA, in c), 9 (Venus, in d), and 11 (eArchT, in d) animals.

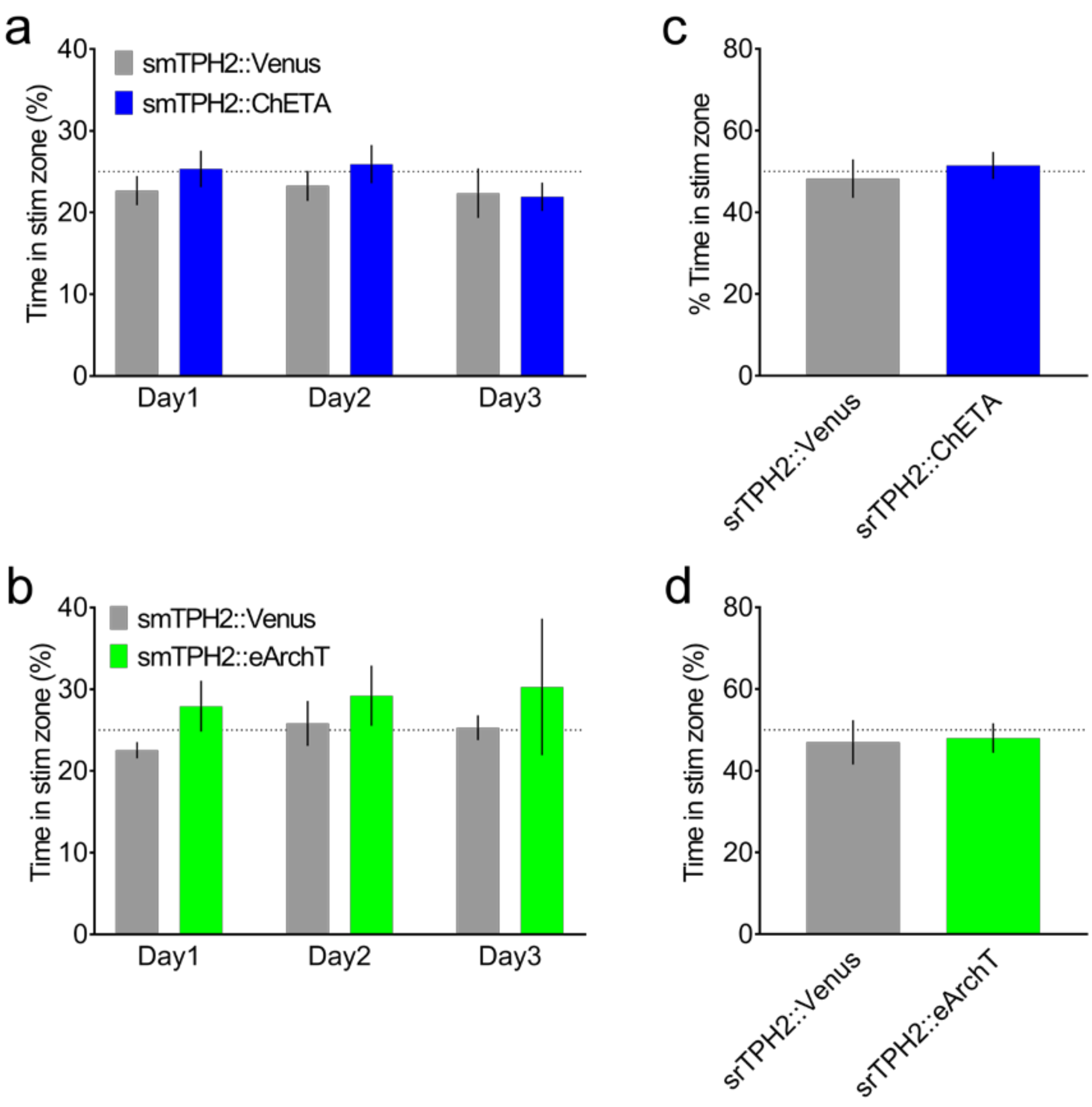

Supplementary Figure S14. Effect of optogenetic activation and inhibition in the real time place preference test.

(a, b) One week after injection of smTPH2::ChETA, smTPH2::eArchT or smTPH2::Venus in the DRN, realtime place preference test was performed for three consective days. Bar graphs show means ± SEM of proportion of spent time in the quadrant associated with blue or green laser stimulation. n = 5 (Venus, in a), 6 (ChETA, Venus in b, eArchT) mice. No significant difference was observed (two-way ANOVA,

Bonferroni's multiple comparison test). (c, d) One week after injection of srTPH2::ChETA, srTPH2::eArchT or srTPH2::Venus in the DRN, realtime place preference test was performed. Bar graphs show means ± SEM of proportion of spent time in the area associated with blue or green laser stimulation. n = 5 (Venus), 6 (ChETA, eArchT) rats. No significant difference was observed (ChETA vs. Venus: $t(9) = 0.5793$, $P = 0.5766$, eArchT vs. Venus: $t(9) = 0.1653$, $P = 0.8724$).

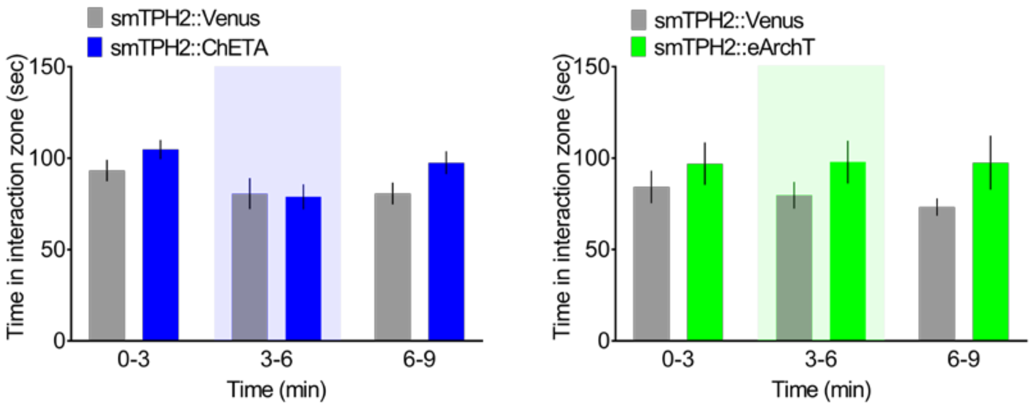

Supplementary Figure S15. Effect of optogenetic activation and inhibition in the social interaction test.

One week after injection of smTPH2::ChETA, smTPH2::eArchT, or smTPH2::Venus in the DRN, social interaction test was performed. Blue or green laser stimulation was applied from 3 to 6 min in the test session. Means ± SEM of spent time in the interaction zone are shown. No significant difference was observed between ChETA- or eArchT-expressing animals and Venus controls. n = 6 mice per group.

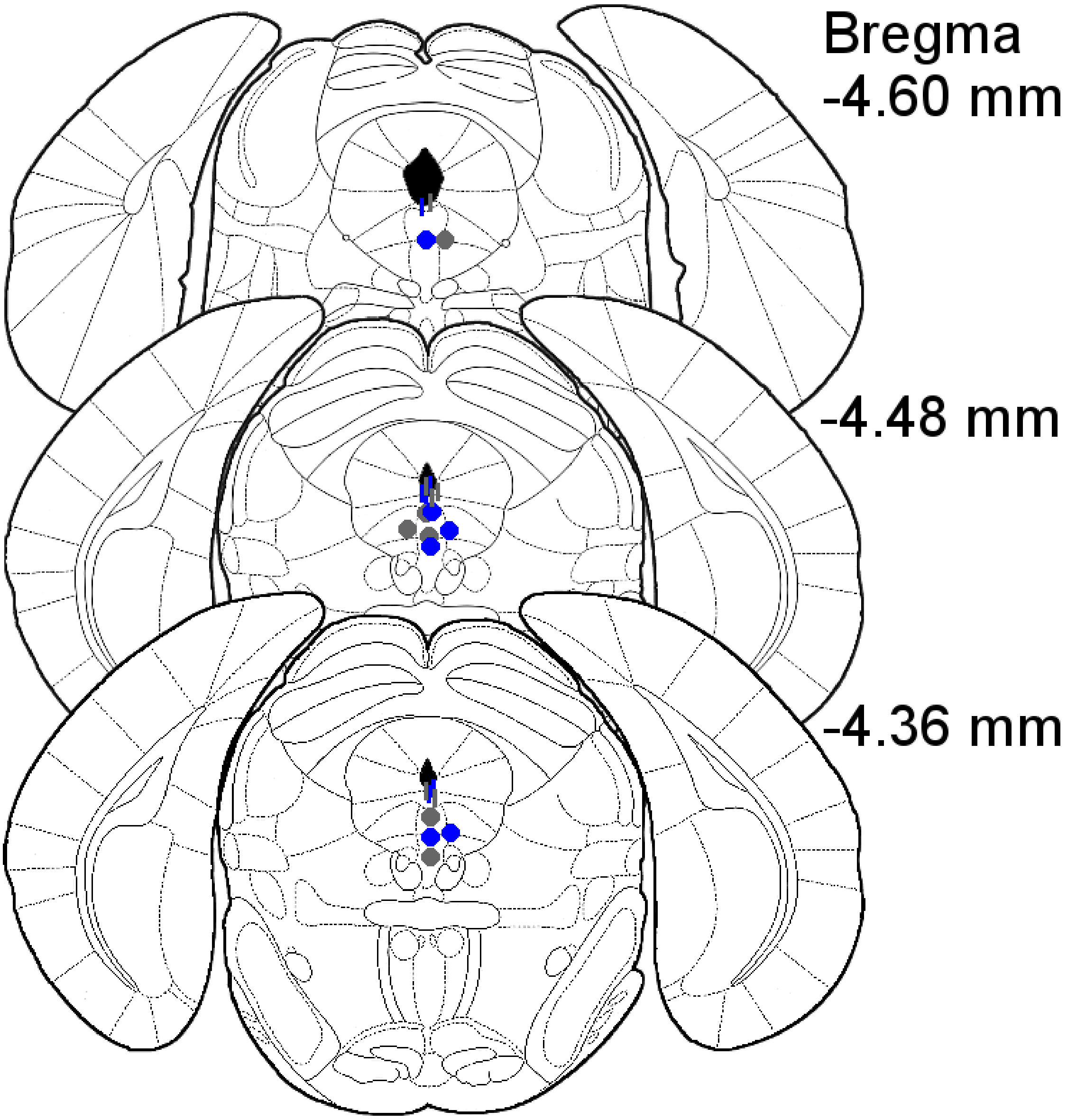

Supplementary Figure S16. Verification of LVV injection site.

After the completion of behavioral analysis, mice injected with smTPH2::Venus or smTPH2::ChETA were sacrificed, and the brain was collected, frozen and sectioned. The LVV injection site was histologically verified under the microscope. Gray circle: smTPH2::Venus, Blue circle: smTPH2::ChETA, Gray line: tip of fiber in smTPH2::Venus, Blue line: tip of fiber in smTPH2::ChETA.

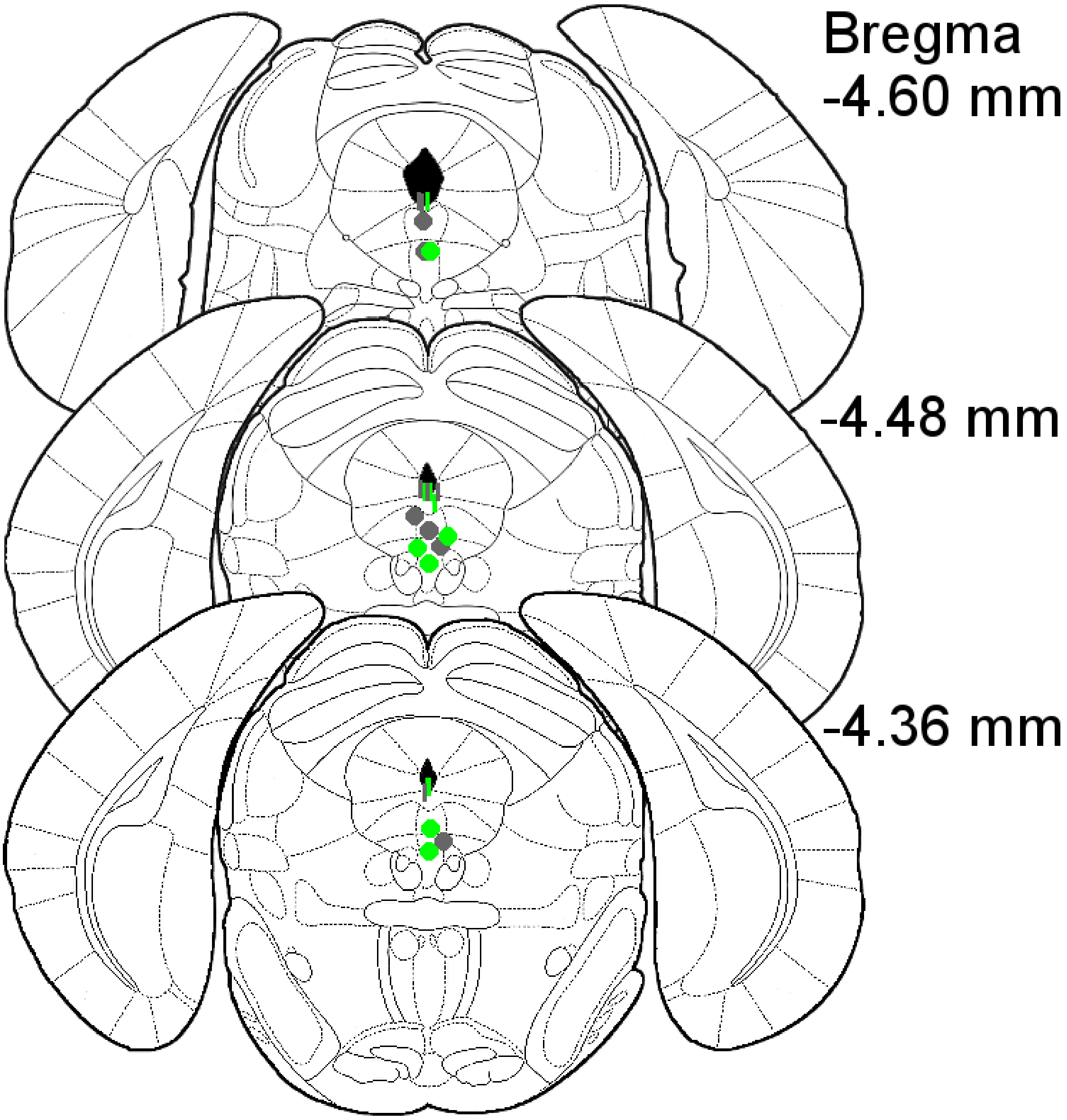

Supplementary Figure S17. Verification of LVV injection site.

After the completion of behavioral analysis, mice injected with smTPH2::Venus or smTPH2::eArchT were sacrificed, and the brain was collected, frozen and sectioned. The LVV injection site was histologically verified under the microscope. Gray circle: smTPH2::Venus, Green circle: smTPH2::eArchT, Gray line: tip of fiber in smTPH2::Venus, Green line: tip of fiber in smTPH2::eArchT.

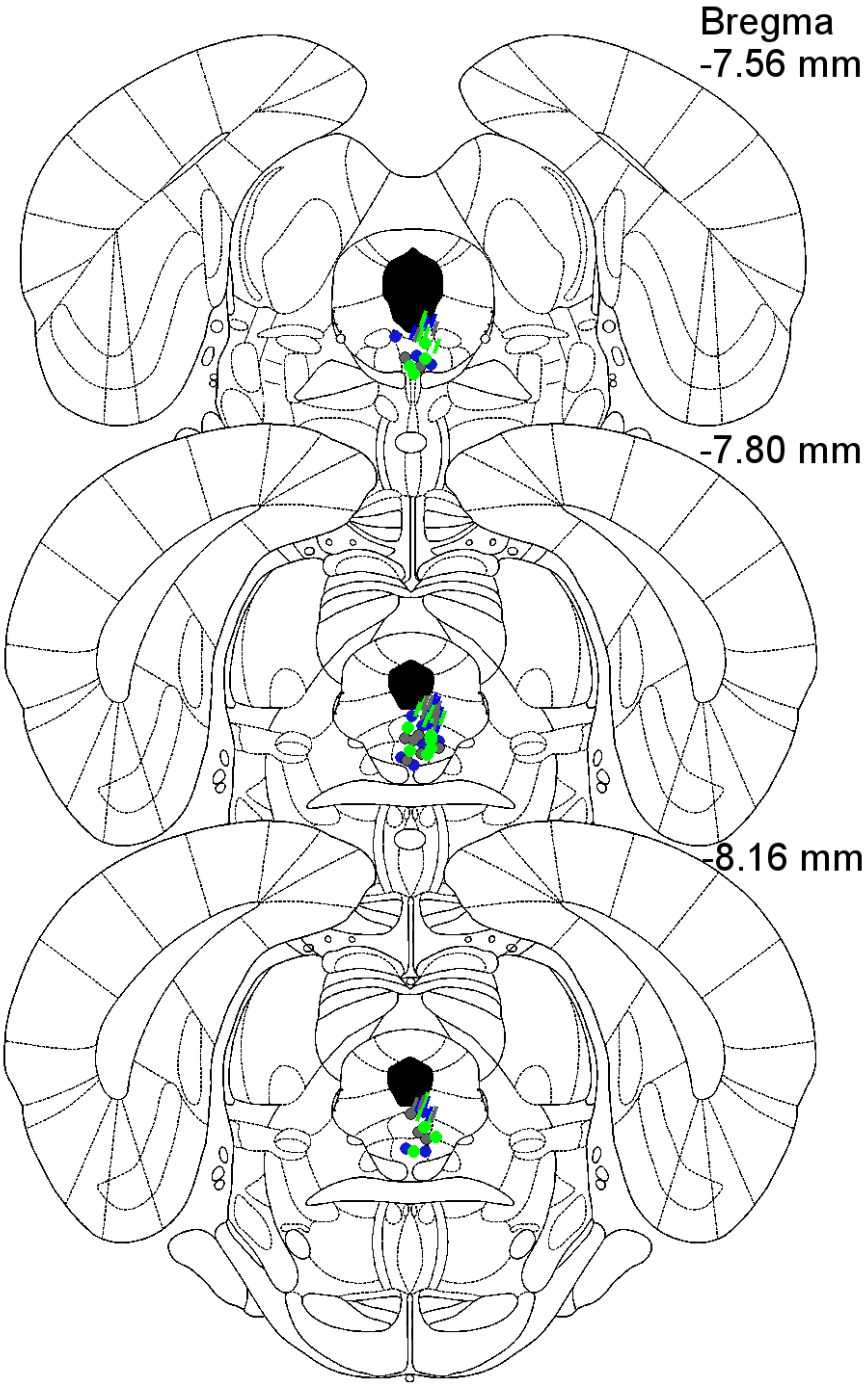

Supplementary Figure S18. Verification of LVV injection site (related to Figure 4).

After the completion of behavioral analysis, rats injected with srTPH2::Venus, srTPH2::ChETA or srTPH2::eArchT were sacrificed, and the brain was collected,

frozen and sectioned. The LVV injection site was histologically verified under the microscope. Gray circle: srTPH2::Venus, Filled blue circle: srTPH2::ChETA, Filled green circle: srTPH2::eArchT, Gray line: tip of fiber in srTPH2::Venus, Blue line: tip of fiber in srTPH2::ChETA, Green line: tip of fiber in srTPH2::eArchT.

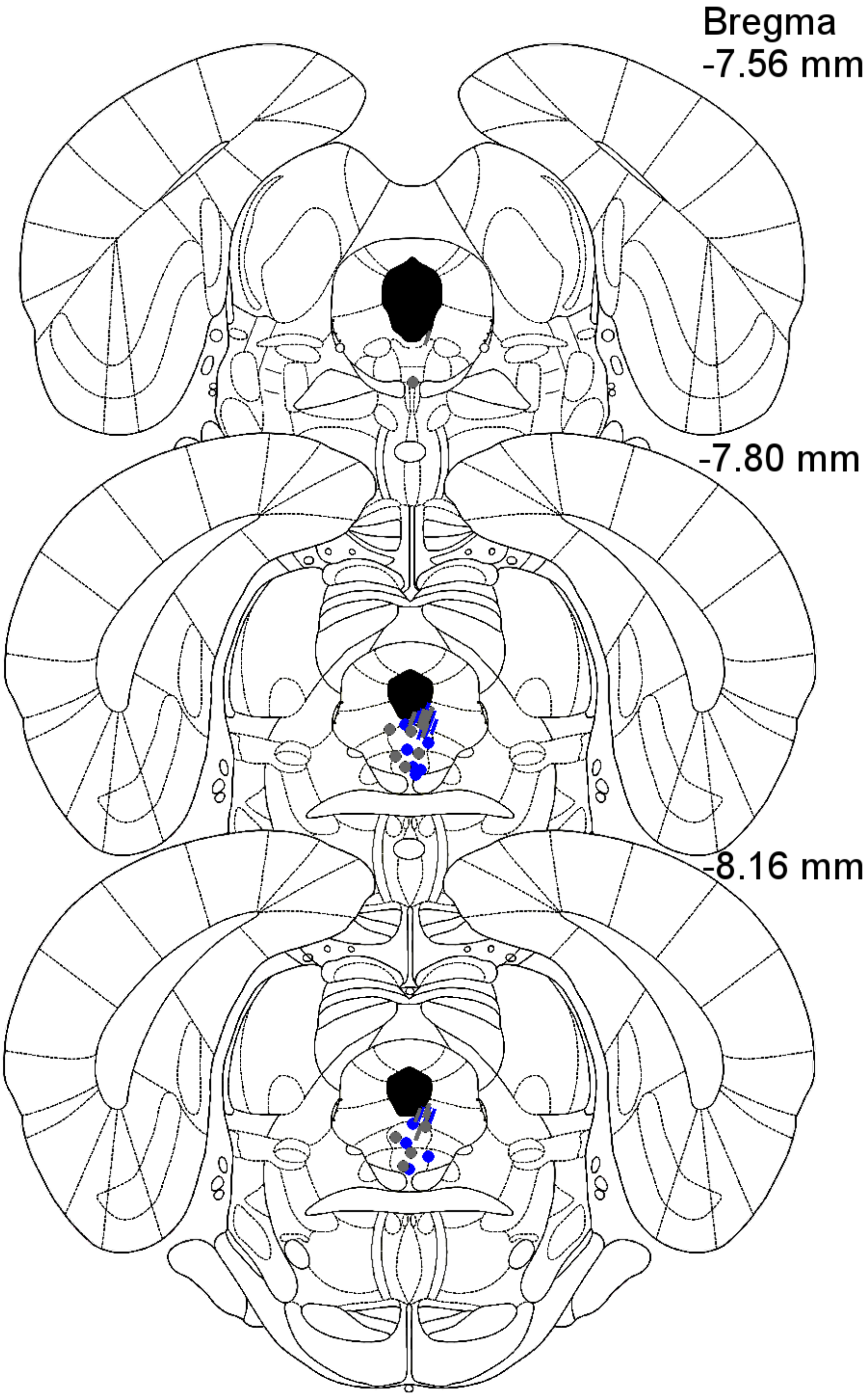

Supplementary Figure S19. Verification of LVV injection site (related to Figure 5).

After the completion of behavioral analysis, mice injected with srTPH2::Venus or srTPH2::ChETA were sacrificed, and the brain was collected, frozen and sectioned. The

LVV injection site was histologically verified under the microscope. Gray circle: srTPH2::Venus, Blue circle: srTPH2::ChETA, Gray line: tip of fiber in srTPH2::Venus, Blue line: tip of fiber in srTPH2::ChETA.

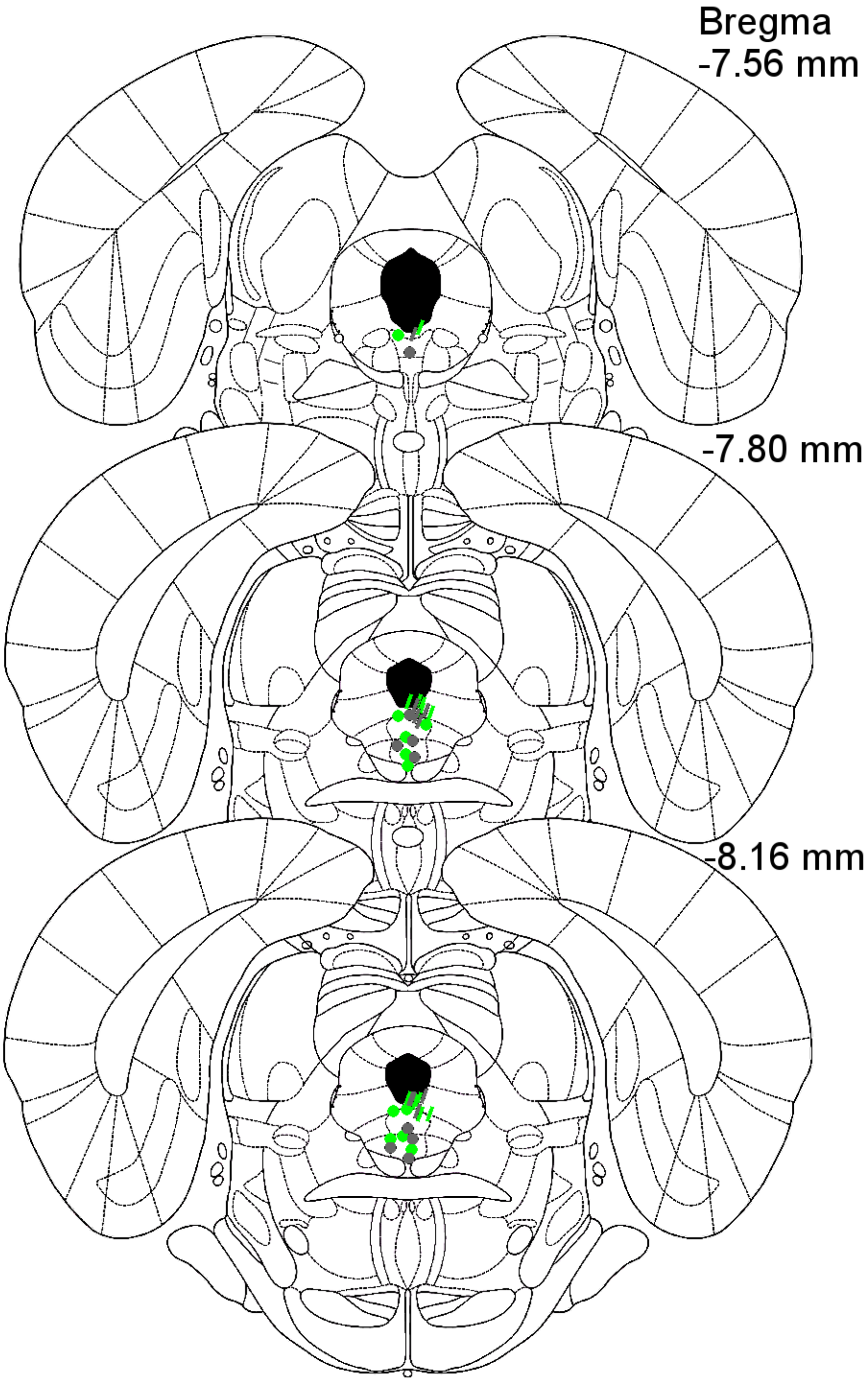

Supplementary Figure S20. Verification of LVV injection site (related to Figure 5).

After the completion of behavioral analysis, mice injected with srTPH2::Venus or srTPH2::eArchT were sacrificed, and the brain was collected, frozen and sectioned. The

LVV injection site was histologically verified under the microscope. Gray circle: srTPH2::Venus, Green circle: srTPH2::eArchT, Gray line: tip of fiber in srTPH2::Venus, Green line: tip of fiber in srTPH2::eArchT.

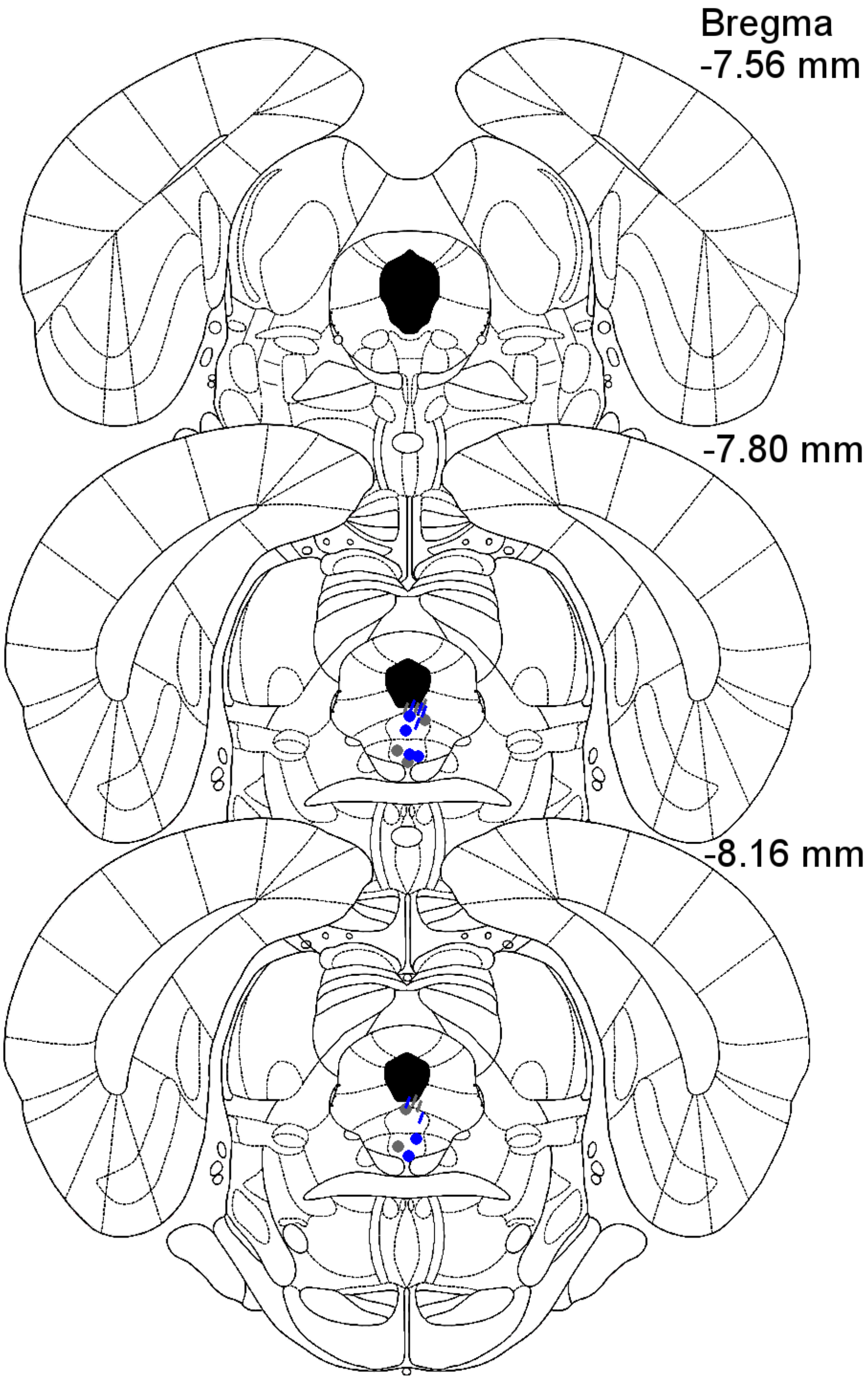

Supplementary Figure S21. Verification of LVV injection site.

After the completion of behavioral analysis, mice injected with srTPH2::Venus or srTPH2::ChETA were sacrificed, and the brain was collected, frozen and sectioned. The

LVV injection site was histologically verified under the microscope. Gray circle: srTPH2::Venus, Blue circle: srTPH2::ChETA, Gray line: tip of fiber in srTPH2::Venus, Blue line: tip of fiber in srTPH2::ChETA.

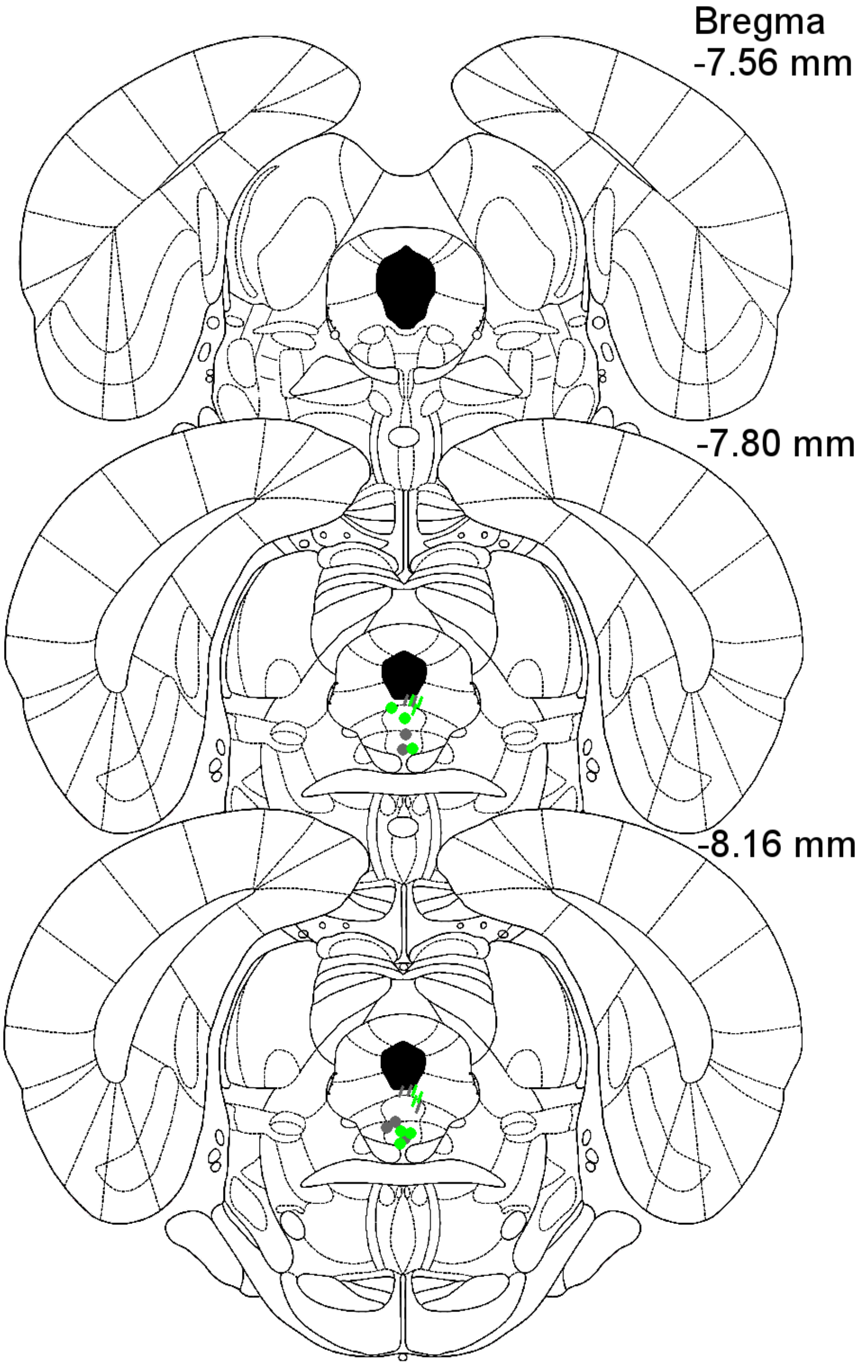

Supplementary Figure S22. Verification of LVV injection site.

After the completion of behavioral analysis, mice injected with srTPH2::Venus or srTPH2::eArchT were sacrificed, and the brain was collected, frozen and sectioned. The LVV injection site was histologically verified under the microscope. Gray circle:

srTPH2::Venus, Green circle: srTPH2::eArchT, Gray line: tip of fiber in srTPH2::Venus, Green line: tip of fiber in srTPH2::eArchT.

Supplementary Table 1.

Oligodeoxynucleotide primer list	
Primer name	Sequence (5' to 3')
NotI Venus Fw	ataGCGGCCGCGGATCCACCATG
SpeI Venus Rv	gagACTAGTTTACTTGTACAGCTC
IRES infusion Fw	TCGGATATCCGTCGAGCCCCTCTCCCTCCCCCC
IRES infusion Rv	AGACAGTAGCTTCATGGTTGTGGCCATATTATCAT
Gal4p65 infusion Fw	ATGAAGCTACTGTCTTCTATC
Gal4p65 infusion Rv	TCCCTCGATGTTAACTTACCTGGTACCGGGCCC
WPRE infusion Fw	GTTAACATCGAGGGATCAAG
WPRE infusion Rv	GTTATCCCTAGTCGACGATGCGGGGAGGCGGCC
NotI Venus Fw2	actGCGGCCGCCACCATGGTGAGCAAG
XbaI Venus Rv2	atgTCTAGATTACTTGTACAGCTCGTC
AscI ChETA-eYFP Fw	aatGGCGCGCCAGCCACCATGGACTATGGC
SpeI ChETA-eYFP Rv	ggcACTAGTTTACTTGTACAGCTCGTCCAT
AscI eArchT-eYFP Fw	aatGGCGCGCCAGCCACCATGGACCCAATT
SpeI eArchT-eYFP Rv	ggcACTAGTTTACACCTCGTTCTCGTAGCA

SacII mTPH2 Fw	attCCGCGGCACTGTAGAAAGCCTTTCCA
MluI mTPH2 Rv	tatACGCGTACTGCAGTGGCAGCTGCCTG
